# Which Compound to Select in Lead Optimization? Prospectively Validated Proteochemometric Models Guide Preclinical Development

**DOI:** 10.1371/journal.pone.0027518

**Published:** 2011-11-23

**Authors:** Gerard J. P. van Westen, Jörg K. Wegner, Peggy Geluykens, Leen Kwanten, Inge Vereycken, Anik Peeters, Adriaan P. IJzerman, Herman W. T. van Vlijmen, Andreas Bender

**Affiliations:** 1 Division of Medicinal Chemistry, Leiden/Amsterdam Center for Drug Research, Leiden, The Netherlands; 2 Tibotec BVBA, Beerse, Belgium; 3 Unilever Centre for Molecular Science Informatics, University of Cambridge, Cambridge, United Kingdom; McGill University AIDS Centre, Canada

## Abstract

In quite a few diseases, drug resistance due to target variability poses a serious problem in pharmacotherapy. This is certainly true for HIV, and hence, it is often unknown which drug is best to use or to develop against an individual HIV strain. In this work we applied ‘proteochemometric’ modeling of HIV Non-Nucleoside Reverse Transcriptase (NNRTI) inhibitors to support preclinical development by predicting compound performance on multiple mutants in the lead selection stage. Proteochemometric models are based on both small molecule and target properties and can thus capture multi-target activity relationships simultaneously, the targets in this case being a set of 14 HIV Reverse Transcriptase (RT) mutants. We validated our model by experimentally confirming model predictions for 317 untested compound – mutant pairs, with a prediction error comparable with assay variability (RMSE 0.62). Furthermore, dependent on the similarity of a new mutant to the training set, we could predict with high accuracy which compound will be most effective on a sequence with a previously unknown genotype. Hence, our models allow the evaluation of compound performance on untested sequences and the selection of the most promising leads for further preclinical research. The modeling concept is likely to be applicable also to other target families with genetic variability like other viruses or bacteria, or with similar orthologs like GPCRs.

## Introduction

### Genetic Information is readily available

Over the last decade extensive sequencing efforts have unraveled the human genome and provide an insight into the extent of human genetic variation [1], [2]. On the one hand this provides possible new drug targets that can lead to new drugs [3]–[5]; on the other hand it shows clearly that natural genetic variation needs to be addressed by some form of personalized medicine that works in a particular patient [6]. An exhaustive, individual “pharmacogenomics” approach for a patient, taking the full genetic make-up of a human into account, is unfortunately not feasible in the short term. This is due to the cost of sequencing but even more so to insufficient understanding of biological processes in humans [7]. However, what is already feasible today for every patient is the full sequencing of pathogens such as bacteria and viruses, as these contain a significantly smaller genome with relatively established locations and functions of drug targets. It is now possible through ‘Deep Sequencing’ technologies, to identify dominant and subdominant viral strains present in an individual patient, paving the way for the development of HIV inhibitors with an optimal potency profile made to target all relevant HIV variants [8], [9].

What is required for the development of an optimal preclinical candidate on the other hand is a knowledge base of the effect mutations have on the binding of current inhibitors. When this information is available it can be used to create a model that allows the user to extrapolate between target sequence variants and predict binding affinities of preclinical compounds on *previously untested* viral target sequences. While similar models have been trained on this data for clinical drugs, these models have in common that they solely are trained on recognizing patterns of the presence and absence of mutations, thus only considering target information [10]–[15]. They do not take into account structural information of the compound – target interaction; hence they are not able to rationalize *why* an inhibitor is active on one sequence but not on another. As a result, the application to the discovery of preclinical candidates is rather limited.

### How to Choose the Right Drug for a Genotype?

In the current work we present one approach to remedy the situation, by making use of the large amount of structural data available on the binding of HIV Reverse Transcriptase (RT) inhibitors to their targets. We will show using prospective experimental validation on hundreds of data points that we can indeed predict which compound is preferable with regard to activity against particular mutants, compared to other compounds. In particular, our aim was to predict activity of compounds on previously *untested* genetic variants of the virus. Given our in-depth understanding of the structural differences between viral enzyme sequences we can incorporate this knowledge to arrive at much improved extrapolation abilities, which enables the design of new inhibitors with improved broad activity profiles.

### Extrapolating in Target Space

When learning from bioactivity data, and attempting to make predictions for novel chemical structures, statistical and machine learning techniques have a proven ability to ‘make sense’ of large data sets under certain conditions (such as interpretable variables used in the model) and to relate chemical structure to activity against a protein target. Bioactivity models are generally based on the ‘Molecular Similarity Principle’ stating that similar compounds (individual compounds or with respect to the distribution of chemistry in a given data set) possess similar properties, such as in this case similar bioactivity [16]–[18]. Yet conventional bioactivity models possess a severe limitation when considering sets of targets, which may be members of a target family such as kinases or G protein-coupled receptors (GPCRs), or as in the case presented here, sequences of viral enzymes. Those models take into account multiple molecules active on a single protein target, yet they completely neglect our extensive knowledge on the similarities of targets to each other. Hence, conventionally a single bioactivity model is generated for every target – neglecting that not only similar compounds show similar bioactivity, but reversely also that similar targets bind similar compounds. In addition, this concept is crucial for the chemogenomics paradigm that has been receiving lots of attention recently [19], [20]. In practice this means that even if a particular ligand-protein target data point is unknown, we can often extrapolate from neighboring activities in both ligand and target space, and not only in ligand space as has previously been done.

In this work we employed a modeling technique called ‘proteochemometric modeling’ (PCM) to model bioactivity data on a set of 14 enzyme sequences. This technique was introduced by Lapinsh, Wikberg *et al.* [21], [22] and similar approaches have since appeared [23]. These techniques have been recently reviewed by the authors [24]. However, no large prospective studies have been presented until today. As PCM uses both ligand and target information, the hypothesis of our work was that PCM would be able to extrapolate the activity of compounds encountered in the training set to novel target sequences. The extension of bioactivity modeling and its impact on preclinical drug research, plus the extensive prospective experimental validation performed, is the main contribution of the current work to the bioactivity modeling field. In addition, applications of PCM to novel target families including, but not limited to: Class A, B and C GPCRs; Kinases; Voltage-gated ion channels and others, can also be covered by this concept. The flexibility of the method is of particular interest when taking current multi-target drug paradigms into account [25], [26].

## Methods

### Data set used to build the models

**Figure 1:**
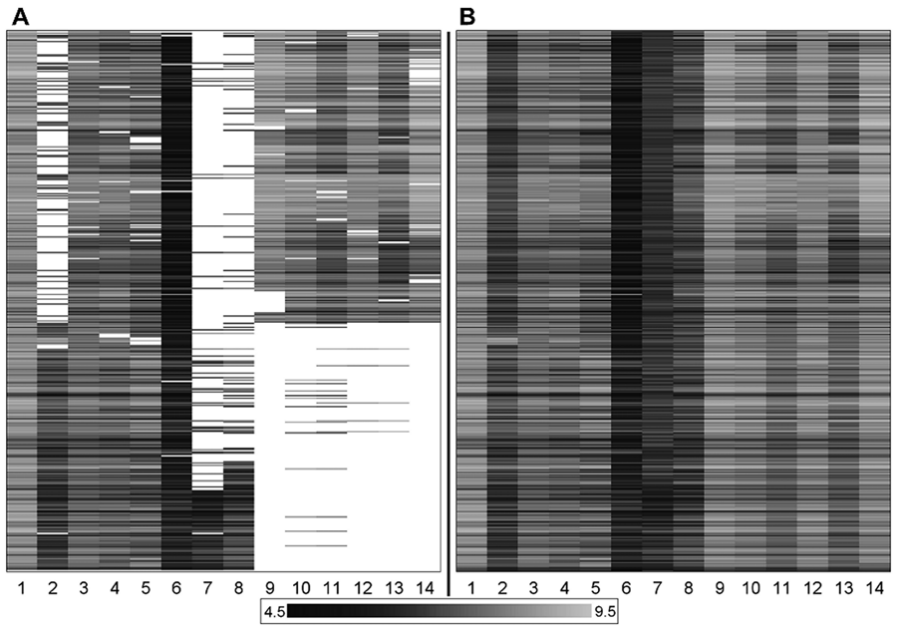
Graphical representation of the NNRTI dataset. (**A**) Our dataset consisted of 451 compounds (in rows) and 14 sequences (in columns) of HIV Reverse Transcriptase with about 60% of the experimental data pairs known. Black indicates a low pEC_50_, grey indicates a high pEC_50_ and white indicates a missing value. (**B**) The dataset with the missing pEC_50_ values completed by our model from which 317 experimental validation data points were chosen.

The data set employed comprises 451 compounds and 14 HIV RT sequences and hence 6,314 possible compound – target combinations. The set was generously provided by Tibotec BVBA. For a total of 4,024 of these combinations an activity value in the form of a pEC_50_ value was available for training the bioactivity model. Compounds with a pEC_50_ value on a certain sequence closer than 0.3 log units to the toxic concentration for that compound (expressed as pCC_50_) were discarded (81 compound – sequence pairs). Table 1 shows the point mutations present in the 13 mutated sequences as well as the wild type (HXB2 / IIIB reference strain [27], sequence 1 in the table) and the average pEC_50_ per sequence. A graphic representation of our data set is shown in Figure 1; a histogram of the pEC_50_ values of all compounds – sequence pairs is available in Figure S8. A sample dataset, the final full model (in the form of a pipeline pilot component), and a protocol to perform PCM are included in the electronic Supporting information.

**Table 1:** Sequence information of the RT sequences in the data set.

Residue/Sequence	89	100	101	102	103	106	118	162	169	179	181	188	190	203	207	210	211	214	215	219	227	234	245	138 (b)	Mean pEC_50_	pEC_50_ (sd)	n
1	A	L	K	K	K	V	V	S	E	V	Y	Y	G	E	Q	L	R	L	T	K	F	L	V	E	8.3	0.6	451
2										F	C														6.9	0.7	259
3											C														7.6	0.6	444
4					N						C														7.5	0.7	443
5		I			N																				7.4	0.8	429
6		I			N					I	C							F						G	6.0	0.6	316
7	S		P	R			I				C		A	V	E	W		F	Y	N			I		6.5	0.6	99
8			P																						6.9	0.7	147
9					N																				8.3	0.6	222
10		I																							7.9	0.7	252
11								K				L													7.5	0.7	257
12			E		N																				8.0	0.6	242
13																					C	I			7.4	0.8	244
14						A			G												L				8.2	0.8	220

Sequence 1 is wild type RT. The other sequences contained one or multiple point mutations of the binding site residues. The mean pEC_50_ of the compounds tested on the sequence is given as is the standard deviation of the series of compounds tested on the sequence. n indicates the number of compounds tested on a particular sequence. Residue number 138 is located on the b-chain of RT while the other residues are located on the a-chain.

### Compound and protein descriptors

All descriptors were calculated in the academic version of PipelinePilot 6.1.5 [28]. Ligands were described by Scitegic FCFP_6 circular fingerprints [29], [30], which have previously been shown to capture a large amount of information with respect to compound bioactivity [31], [32]. FCFP_6 descriptors provide individual substructures and treat these as a feature of a compound. These substructures have a maximal diameter of 6 bonds from a central atom. In the final model we can link these substructures to a change in pEC_50_.

**Figure 2:**
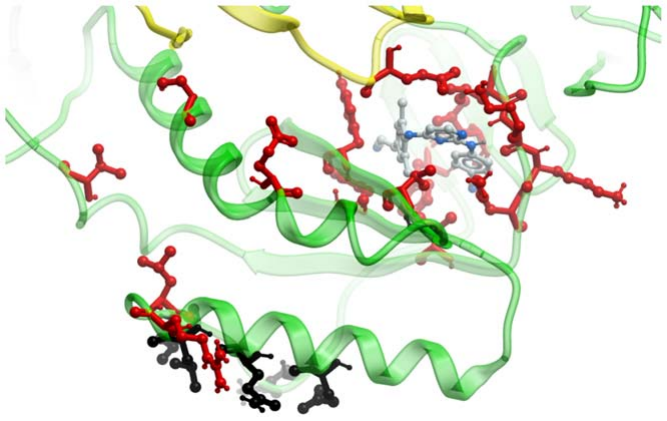
The binding site used in our models. PDB X-ray structure 2ZD1 of RT with Rilpivirine, an NNRTI like the analog series we modeled and shown in grey. The residues that were used to identify the binding site (and which were used for activity modeling) are shown in black and red. The black residues are only mutated in sequence 7 (heavy mutant) while the red residues are mutated in more than one sequence. Indicated by a green ribbon is the ‘A’ chain of HIV RT while the ‘B’ chain is displayed in yellow.

Sequences were encoded based on the binding site sequence in which each amino acid was represented as a single unique feature. The residues used to define the binding site are shown in Figure 2 (PDB Code 2ZD1, HIV RT bound to Rilpivirine [33], created with Molsoft ICM version 3.6-h) and Table 1. The residues used to define the binding site are shown in red and black, where the black residues are the ones that were mutated only in sequence 7. The features describing the binding site were obtained by hashing an array of 58 physicochemical properties obtained from the AAindex database [34]; the used indices can be found in Table S3.

Finally, both ligand and protein fingerprints were converted to a fixed length array of features which were then used in the modeling. The ligands were converted to a fixed length array of 155 features while the sequences were converted to a fixed length array of 24 features using default Pipeline Pilot settings.

### Machine learning

Models were constructed in the academic version of Pipeline Pilot 6.1.5 [28] using the R-statistics package. Support vector machines (SVM) as coded in the e1071 package were used for model creation [35]. Parameters gamma and cost were tuned over an exponential range and epsilon was set at 0.2 as this was the given assay error. It has been shown that setting epsilon to the data error is the optimal value for training [36]. The optimal model was determined using 5-fold cross validation before proceeding to experimental prospective validation of the model. The parameters used for validation were RMSE and R_0_^2^, although the regular R^2^ was also determined it has been shown that R_0_^2^ provides better reliability [37], [38]. R_0_^2^, in contrast to R^2^, takes into account that the regression line of our model predictions should intersect with the origin. *In silico* validation on trained models was done via learning curves (see Figure S9).

### Prospective Experimental Validation

To assess the prospective capabilities of PCM we used our final model, which was trained on the full data set, to predict the activity of all compounds for which no pEC_50_ value was available. In total 835 data points were subsequently experimentally validated, 317 of these represented novel predictions and 518 were repeat experiments to establish reproducibility of the assay. The 317 novel predictions included 130 compound – sequence pairs that were predicted to differ 2 standard deviations from either compound average (69 compound – sequence pairs), called *compound outliers*, or sequence average (61 compound – sequence pairs), called *sequence outliers*. Therefore we specifically tested the ability of our model to extrapolate outside of the bioactivity space of ‘typical’ compound – sequence pairs.

The techniques used as a benchmark were QSAR, kNN and pEC_50_ scaling. Individual QSAR models were trained for all sequences based on the same FCFP_6 compound descriptors as the PCM model. The kNN models were created based on Jensen *et al.* [39]. In total nine kNN models were created. Three models were based on only compound data using 3, 10 or 20 neighbors, three models on only target data using 3, 10 or 14 neighbors and three models based on both compound and target data using 3, 10 or 20 neighbors. In addition we also performed simple interpolation of pEC_50_ values (‘scaling’), by assuming that the affinity of a compound on a sequence follows the general trend displayed by a series of other compounds. Thus, if a series of compounds on average has a 0.3 log unit lower pEC_50_ value than on the wild-type sequence, then the new compound is also assigned a 0.3 log unit lower pEC_50_ value.

### Antiviral assays

The antiviral activity of different inhibitors was determined in a cell-based HIV-1 replication assay. Here MT4 cells (150,000 cells/ml), stably transformed with an LTR-EGFP reporter gene, were infected with HIV-1 (IIIB, clinical isolates, or site-directed sequence strains; multiplicity of infection MOI = 0.0025) in the presence or absence of different inhibitor concentrations. After three days of incubation, the amount of HIV replication was quantified by measuring the EGFP fluorescence, and expressed as EC_50_ values. The toxicity of inhibitors was determined in parallel on mock-infected MT4 (150,000 cells/ml) cells stably transformed with a CMV-EGFP reporter gene and cultured in the presence or absence of test compound concentrations. After three days of incubation, cell proliferation was quantified by measuring the EGFP fluorescence, and expressed as CC_50_ values (cytotoxicity, 50% inhibitory concentration of cell growth).

### Leave-one-sequence-out validation

In order to assess the models’ capability to extrapolate between the different sequences we performed ‘Leave-one-sequence-out validation’ (LOSO). In this approach for each of the 14 sequences all data points of the sequence under consideration are left out of the training set, a model is trained on the remainder and the points left out are used as a test set for that model. Both the RMSE and the R_0_^2^ values of the validation plot were subsequently determined for each of the 14 sequences in turn.

### Model interpretation

To determine the effect of individual residues, for each sequence each residue was mutated back to wild type *in silico*. Subsequently for all compounds the model prediction on the original mutant sequence was compared with the prediction of the model on the *in silico* changed mutant sequence. The difference was interpreted as the change in pEC_50_ induced by that particular residue. From all these changed prediction values the following values were calculated: the average overall, the average per sequence and per individual mutation. This provided the model interpretability. However, changes that led to a 0 value shift in pEC_50_ were removed in the calculation of the average per position since in all cases this was caused by mutation back to wild type of a residue that was already wild type in that particular sequence. In addition the large amount of 0 value shifts lead to a shift of the average towards 0 when it was calculated thereby masking the actual contribution of mutations.

To interpret the influence of compound substructures a slightly different approach was chosen. Here each FCFP_6 feature, corresponding with a particular substructure, was substituted by the feature representing a single Carbon atom (‘0’). Since this carbon atom was already present in all compounds, its effect on binding serves as a calibration. Subsequently the full model was used to predict the pEC_50_ of the adapted compound fingerprint lacking a certain substructure on all sequences and compared with the model prediction of the original compound fingerprint on all sequences. From all these changed predictions the average overall and per sequence was calculated for that particular feature, or chemical substructure in this case.

### Chemistry

Synthesis of the analog series we used in this work has been described in multiple patents. Compound 1 is listed in patent WO 2007/113256 [40], compound 2 is listed in WO 2008/080965 [41] and compounds 3 and 4 are listed in WO 2008/080964 [42]. Compound 6 is listed in patent WO 2008/080965 [41], compounds 5, 7 and 8 are published in WO2007/113254 [43]. In addition the electronic Supporting information to this work contain the structures of 57 of the modeled compounds. For these compounds the full biological activity spectrum on the 14 sequences as we had it available is included along with the pEC_50_ predicted by our model on each of the 14 sequences (Table S4 and Archive S1).

## Results and Discussion

In the current work PCM was applied to a data set of Non-Nucleoside Reverse Transcriptase Inhibitors (NNRTIs), which constitutes one of the major classes of anti HIV drugs on the market. Among these are compounds such as Nevirapine [44] and Efavirenz [45], but also novel compounds such as Etravirine [46], which was approved by the FDA as recently as 2008. Since NNRTIs are allosteric binders they have shown considerably fewer side effects than orthosteric drugs [47]. However they are also known for a quick onset of viral resistance due to the accumulation of resistance associated mutations [48], [49]. Hence, the pharmacological profile of NNRTIs is highly desirable, but their effectiveness is hampered by the onset of resistance. This problem is also encountered in other viral infections like Hepatitis B [50], Hepatitis C [51] and Influenza A (H5N1) [52], [53]. Resistance can therefore be considered a universal problem when developing a new anti-viral drug. Thus, when new anti-virals are developed it is important that they retain their effectiveness despite the presence of these mutations. To be able to predict the activity of a preclinical drug candidate on an adapted pathogen would be an important contribution to drug discovery. Here we present an application of PCM that can predict drug performance on unknown sequences or adapted pathogens, when staying within model limitations.

### Solving the problem of sparse data sets

Our data set contains compounds that inhibit wild type Reverse Transcriptase (RT), but also some that inhibit a number of RT sequences that are highly resistant against inhibition. NNRTIs are allosteric inhibitors of RT, which is illustrated in Figure 2 [33]. Incomplete bioactivity data sets are common in real-world settings, and this study is no exception. The data set is graphically displayed in Figure 1, with ‘blanks’ representing unknown data points that we would like to predict using computational methods. A pEC_50_ value was available for approximately 60% of the data set.

In a preclinical drug discovery setting, the ability to make decisions on a full rather than a sparse matrix increases the likelihood that the best candidate will be selected. This is of vital importance as a SAR table of drug – target interactions does not necessarily show linear relationships, e.g., a substituent in a given compound will not lead to the same increase or decrease in binding on different targets. This is even the case in analog series like our data set [54], [55]. Especially in anti-viral research compounds can display unexpected behavior on the different sequences. It is this behavior we are able to capture and translate into accurate predictions.

### Prospective Experimental Model Validation

During model development, learning curves were generated that represent *in silico* validation, in addition we performed Y-scrambling [56] (Figure S9 and Figure S11). More importantly, we predicted the pEC_50_ of 317 unknown compound – target pairs. These predictions were subsequently measured experimentally. By predicting first and subsequent experimental validation, we obtain an estimate of model performance when considering novel compound – target pairs. Here we explicitly selected the more ‘difficult’ compound – target pairs; namely those with predicted pEC_50_ values that are either atypical for the particular compound tested (compound outliers), or for the particular sequence under consideration (sequence outliers). It is trivial to pick the compounds that are always active or always less active – we precisely removed those cases from our prospective testing and focused on the inhibitors that *were predicted to be active against highly resistant sequences*, *or those which were predicted to be less active against very susceptible sequences*.

**Figure 3:**
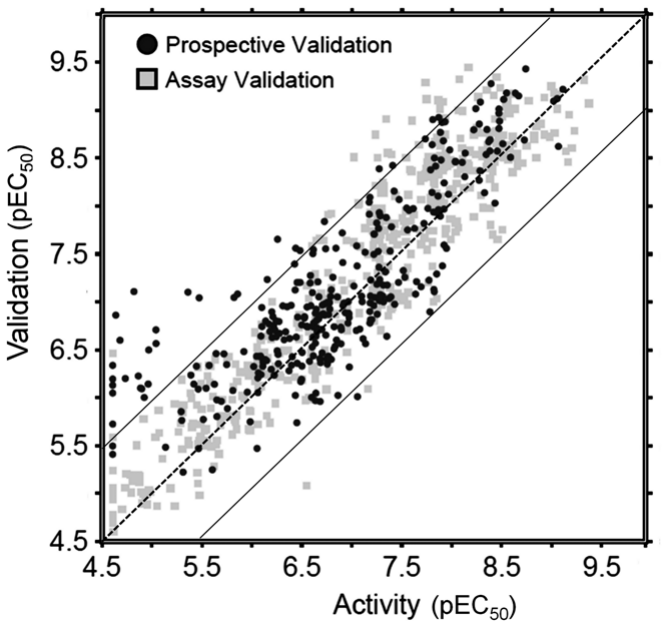
Model performance in the prospective experimental validation. Shown are both predictive performance of the model (black dots; R_0_^2^: 0.69, RMSE: 0.62 log units) and assay reproducibility (grey squares; R_0_^2^: 0.88, RMSE: 0.50 log units). The continuous lines indicate an error of 1 log unit, while the center dashed line indicates a perfect correlation (see main text for further details).

Figure 3 shows the performance of the model, trained on the full data set, in these prospective validation experiments. During cross-validation the full model achieved a Root-Mean-Square Error (RMSE) of 0.38 log units (with a Q^2^ of 0.84) while employing 155 ligand features and 23 protein features. When applied to the new untested data, the model achieves an RMSE of 0.62 log units and an R_0_^2^ of 0.69 in the prospective validation. Our model can predict the pEC_50_ of *untested* compound – target pairs with average accuracy of 0.62 log units. This RMSE approaches the reproducibility of the assay which was 0.50 log units (R_0_^2^ of 0.88).

To benchmark this performance against conventional approaches we applied QSAR modeling, k-Nearest Neighbor (kNN) modeling (using 3, 10 or 20 nearest neighbors and based on compound, target, or compound and target information) and pEC_50_ scaling to the same data set. The results are shown in Table 2. Here PCM outperforms QSAR and all forms of kNN modeling, while pEC_50_ scaling seems to perform slightly better. More specifically, PCM had an RMSE of 0.62 log units, while kNN showed 0.90 for the best model and scaling performed slightly better with 0.57. The R_0_^2^ reached 0.69 for PCM, where kNN showed 0.41 and scaling also reached 0.69. Scaling of pEC_50_ values performs second best but this method has two major disadvantages. When we consider the sequence and compound outliers, PCM outperformed the simple pEC_50_ scaling. It is precisely those data points that points are most interesting in research. The sequence outliers represent the inhibitors that inhibit all present HIV mutants, *candidates to select in lead selection*. The compound outliers can be completely inactive on one particular sequence, *candidates to avoid in lead selection*. When considering these compounds, the average RMSE was 0.52 for PCM and 0.58 for scaling (R_0_^2^ was 0.61 for PCM and 0.59 for scaling). A second disadvantage of scaling is that this method cannot be applied to untested viral sequences, whereas PCM can.

**Table 2:** Performance of different methods in experimental validation.

ValidationExperiment	Assay	PCM	pEC_50_scaling	QSAR	3-NN(both)	3-NN(target)	3-NN(cmpd)	10-NN(both)	10-NN(target)	10-NN(cmpd)	20-NN(both)	14-NN(target)	20-NN(cmpd)
R_0_^2^ (Full plot)	0.88	0.69	0.69	0.31	0.38	0.04	0.21	0.41	0.21	0.28	0.40	0.21	0.28
RMSE (Full plot)	0.50	0.62	0.57	0.96	0.90	1.55	1.24	0.90	1.29	1.16	0.90	1.21	1.17
R_0_^2^ (Sequence Outliers)	0.88	0.65	0.52	0.32	0.32	<0.00	<0.00	0.32	0.15	0.03	0.32	0.22	0.02
RMSE (Sequence Outliers)	0.50	0.39	0.52	1.40	0.57	2.25	1.68	0.57	1.88	1.65	0.57	1.79	1.67
R_0_^2^ (Compound Outliers)	0.88	0.56	0.65	0.39	0.30	0.19	0.18	0.36	0.49	0.33	0.38	0.54	0.35
RMSE (Compound Outliers)	0.50	0.65	0.64	0.72	0.87	1.18	1.05	0.86	0.90	0.93	0.86	0.82	0.92
R_0_^2^ (Outliers)	0.88	0.61	0.59	0.36	0.31	<0.00	0.08	0.34	0.32	0.18	0.35	0.38	0.19
RMSE (Outliers)	0.50	0.52	0.58	1.06	0.72	1.72	1.37	0.72	1.39	1.29	0.72	1.31	1.30

PCM and pEC_50_ scaling outperform the other techniques (QSAR and k-Nearest Neighbors) with pEC_50_ scaling having a slight advantage. However, PCM performs better when the selection is narrowed to those compound – sequence pairs that show a pEC_50_ two standard deviations higher or lower than average (shown here as Sequence and Compound Outliers). Also shown is the average score of each technique on the combined outliers (shown as Outliers). Negative values are denoted as <0.00.

Hence, we conclude that the prospective experimental validation confirms the validity of our model, and the applicability of PCM to extrapolate in both ligand (chemical) space and target (biological) space. The other benchmarked techniques, kNN and QSAR, do not accurately capture the compound – target interaction space. The ability to model all these situations with a comparable reliability and the possibility to interpret the model from both a sequence and compound perspective, are the major advantages of PCM over kNN, QSAR and scaling methods.

### Neighborhood Behavior in Target Space

In the prospective validation we noticed that a number (15) of data points were predicted inaccurately with an error larger than twice the RMSE, all of which involved sequence 7. Trying to elucidate the reason for this behavior the different sequences were clustered based on similarity (Figure S5 for details), where sequence 7 was found to be most dissimilar to all other sequences, containing a rather large number of 13 point mutations (for sequence information see Table 1). In addition, it is the sequence with the smallest number of tested compounds in the training set, thus diminishing the number of compound – target pairings that were used in the model directly (without extrapolation from neighboring sequences), thereby rationalizing the large number of false predictions on this sequence.

**Figure 4:**
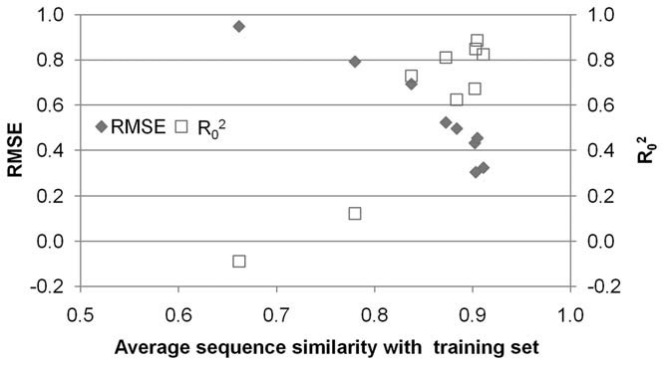
Extension of the applicability domain to target space. Prediction error (measured as R_0_^2^ and RMSE in log units) versus the average similarity of the sequence to the rest of the training set, extending the ‘Molecular Similarity Principle’ to biological space which is of crucial relevance in PCM. It can be seen that a higher similarity to the training set leads to more accurate predictions. Still, predictions on the most dissimilar sequence have an average error of less than 1 log unit.

In order to systematically investigate the dependence of the prediction error on the sequence similarity we plotted the prediction error as a function of the average Tanimoto similarity of the individual sequences to the rest of the training set (shown in Figure 4). A correlation between model error and average sequence similarity is observed. The prediction error increases when the average similarity decreases between the sequence, for which the compound activity is predicted, and the rest of the training set sequences. This observation is an extension to the ‘Molecular Similarity Principle’ which states that similar compounds have similar properties, and in this work we are able to show that this paradigm also holds in biological space where *similar sequences show similar ligand binding abilities*; a concept we are now able to quantify numerically. This extends recent work on ‘applicability domains’ and ‘activity cliffs’ by also taking the biological target side into account [54], [55], [57], [58]. An improved distance measure is an ensemble of both the distance between the compounds (Figure S10), as has been previously shown [16], and the distance on the *target side*. To our knowledge this neighborhood behavior in target space has not been previously shown but is a natural extension of the *chemical similarity principle* to this new technique.

The dependence of model accuracy on the average distance provides a useful tool to set the model applicability domain. As this distance is a property dependent on the training set on one hand and the unknown compound – target pair on the other hand it can be measured before any model prediction is made. Furthermore, neighborhood behavior can determine beforehand if the model is capable of predicting pEC_50_ changes on a previously untested genotype. This approach ensures that only model predictions with certain accuracy are used and that those that do not meet this accuracy are disregarded. For our current model this accuracy is defined as an error in pEC_50_ prediction (Figure 4) but this can also be an error in pKi value or any other value the model is trained to predict.

### How to anticipate bioactivity for *novel* protein targets?

As we have shown that our model displays neighborhood behavior in target space, here we explore the possibility to use such a model in extrapolation. Conventionally a fraction of the full data set is left out from the training set when testing bioactivity models. The ability of the model to make predictions for the previously untested (‘novel’) compounds is taken as a predictor of model performance. In our case, we not only extrapolated in *compound space*, but also in *sequence space*. Hence, in order to confirm the ability of the model to extrapolate the activity of compounds to related sequences we performed a ‘Leave-one-sequence-out’ experiment (LOSO). Here, it emulates the prediction of inhibitor activity for a virus with a *not previously* encountered RT sequence, based solely on bioactivity measurements against other sequences in the data set. While applied to enzyme mutants here, the concept is generally applicable and the authors are currently investigating its performance on other target families such as GPCRs.

**Figure 5:**
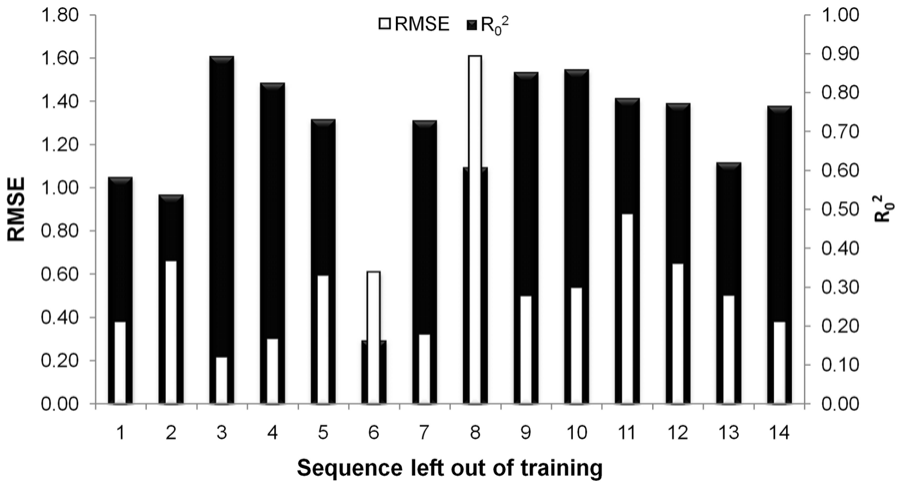
Performance of PCM in leave-one-sequence-out experiments. Performance was measured by R_0_^2^ and the RMSE in log units. The number below the bar corresponds to the sequence left out of the training set.

Figure 5 shows the R_0_^2^ and RMSE of the LOSO validation experiment (see Figures S1 and S2 for additional information including error depending on similarity to the training set in sequence space). We observe that for 13 of the 14 sequences an RMSE of less than one log unit was obtained, and five of the 14 sequences even yielded an RMSE of less than 0.5 log units, which is the order of magnitude of assay reproducibility. Most interestingly, the model was able to use the information contained in all the different mutant sequences and predict the affinity of the compounds on the wild type sequence (Sequence 1). This means that this LOSO-1 model was able to deconvolute the individual contributions of the different mutants. Furthermore other LOSO models were also capable of predicting activity on unknown sequences 2–5 and 9–14. All these sequences contain very different mutations (Table 1). Finally, the LOSO-7 model was able to predict the pEC_50_ of the compounds on the “heavy” mutant (sequence 7), which contains a total of 13 point mutations. These findings underline the ability of PCM to extrapolate in target space, which supports the application of the technique to predict compound activity on different mutants.

Our results show that the model is indeed able to predict the pEC_50_ values of a known compound on an unknown sequence. However it should be noted that PCM performs lesser in 2 of the 14 mutants considered. The first of the two exceptions is sequence 6, which can be seen as a singleton since one of the mutations it carries (E138G) is only present in this particular sequence. Therefore, bioactivity prediction based on other sequences that do not carry this mutation is not straightforward. It was already known from the full model interpretation (see ‘Model Based Interpretation of Mutants’) that the impact of this mutant was underestimated. The LOSO-6 model correctly predicted that all compounds have a lower activity on this sequence (giving rise to a small RMSE value); however, the ranking among compounds is not very accurate (explaining the low R_0_^2^).

The second sequence where our model underperforms is sequence 8. This case is different from the previous one: while the model is able to correctly rank the compounds, leading to an acceptable R_0_^2^ of about 0.6, it consistently overpredicts the activity of compounds, leading to a high RMSE. In sequence 8 at position 101 a positively charged lysine residue is replaced with a proline, which likely induces conformational changes to the backbone of the protein. K101P is present as a single mutation in sequence 8, as well as in combination with a total of 12 other mutations in sequence 7 (which is then the only sequence that contains K101P in the training set when sequence 8 is left out for LOSO validation). Hence, the model likely underestimates the impact of the K101P mutation due to the large number of other mutations present in sequence 7; it is not able to deconvolute the impact of every single mutation properly. In this case, while the model would overestimate drug activity on this particular target, in a computer-aided compound selection setting the correct candidate would still be identified due to the accurate ranking of the compounds by the model.

### Model performance in relation to chemical structure

**Figure 6:**
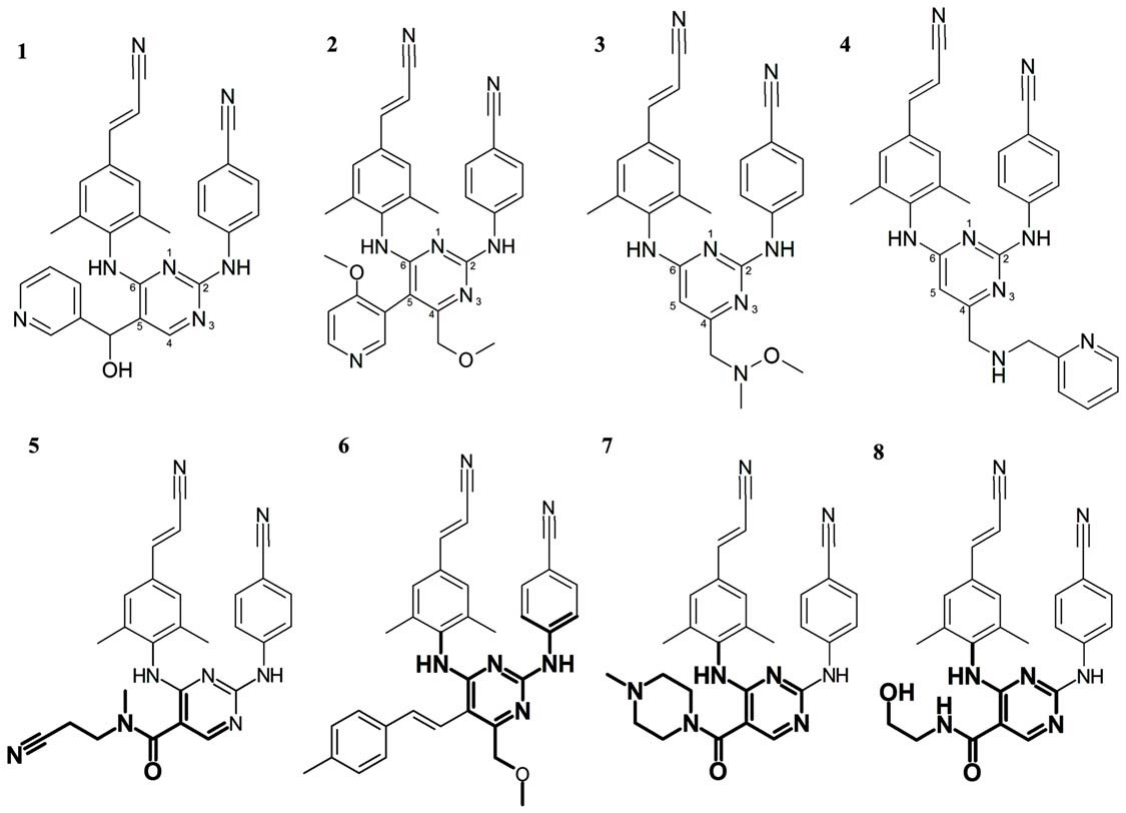
Example structures that where included in the model. A selection of both compounds containing accurately (**1,2**) and inaccurately modeled chemistry (**3,4**). Also shown are compounds containing a substructure positively correlated with pEC_50_ (**5,6**) and compounds containing a substructure negatively correlated with pEC_50_ (**7,8**). In the upper part, shown are sample compounds that were accurately predicted using the LOSO models, a low RMSE (**1**, RMSE was 0.22 log units, R_0_^2^ was 0.96) and a high R_0_^2^ (**2**, R_0_^2^ was 0.89 , RMSE was 0.26). Secondly, sample compounds that were predicted inaccurately using the LOSO models, a high RMSE (**3**, RMSE was 1.12 log units, R_0_^2^ was −0.10) and a low R_0_^2^ (**4**, R_0_^2^ was −3.66, RMSE was 0.61). The lower part shows the 17^th^ best substructure (**5**) and the 30^th^ best substructure (**6**). Conversely the 3^rd^ worst substructure (**7**) and the 4^th^ worst substructure (**8**) are depicted.

To get an idea of the ability of the LOSO models' ability to predict individual compounds we have shown representative examples of compounds either predicted accurately or inaccurately in Figure 6. The activities of compounds **1** and **2** on the different sequences were correctly predicted by our leave-one-sequence-out models, while compounds **3** and **4** were predicted inaccurate (see Figures S3 and S4 for the predictions and experimental activities of the compounds discussed here). Please note that we made predictions challenging as we used LOSO models to predict the activity of compounds on the sequences left out of the training of these exact LOSO models. It is therefore a realistic emulation of a preclinical PCM application.

From the data we conclude that inaccurately predicted compounds have a large functional group in the 4 position of the pyrimidine ring. Apparently the LOSO models are unable to capture this information correctly. When we further analyzed the individual predictions on the different mutants (shown in Figure S4), we noticed that the compounds are predicted accurately (error <0.5 log units) on the majority of the sequences. The underperformance for compound **3** is caused by overprediction on sequence 8 (2.7 log units, carrying K101P) and the underperformance of compound **4** by underprediction on sequence 2 (carrying V179F).

To explain this behavior, we need to consider the ligand binding mode. A shared binding mode of all compounds is very likely since i), they all share a common chemical scaffold and ii), NNRTIs are known to have a highly homologous binding mode [59]. Hence we can correlate these mispredictions with the protein structure. The substitution position on the pyrimidine ring in the compounds corresponds to the location of residues L100, K101 and V179 in crystal structure 2ZD1. It is known that these residues mutate easily and sequences carrying point mutations in this location are present in our training set [60]. When studying the crystal structure, it can be seen that the side chains of these residues are likely to hamper the binding of compounds with a large functional group in position 4.

Hence, we propose the presence of a large functional group on position 4 on the pyrimidine ring to be a predictor of insufficient model performance in combination with mutations on positions 101 and 179 in the protein. While compounds *with* a large substituent on the 4 position are accurately predicted on targets *not carrying* mutations on positions 101 and 179, they tend to be predicted inaccurately when the targets *are mutated* in these positions. However, if no large substituent is present on the 4 position, compounds *are* predicted accurately on targets carrying mutations on positions 101 and 179. Using this knowledge we can define an applicability domain when applying this particular model, but this finding should also be taken as a warning when using any PCM model to predict the affinity of known compounds on unknown sequences.

### Model based interpretation of mutations

**Figure 7:**
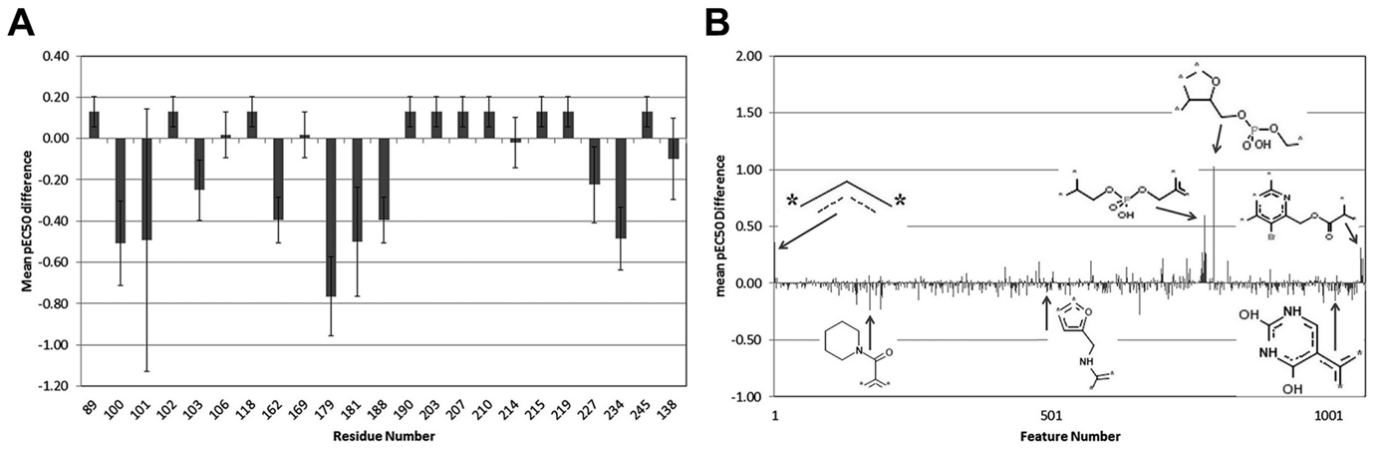
Interpretation of the full model. (**A**) Overview of the contribution of mutations present at all individual residue positions. The full model explains the lowered activity (pEC_50_ in log units) on mutants mainly by mutations at residues 100, 101, 103, 162, 179, 181, 188, 227, 234 and residue 138 on the ‘b’ chain. (**B**) Overview of the contribution of the different chemical substructures. Substructures occurring only in a single compound have been removed and the remaining substructures have been numbered sequentially. Several substructures have been visualized and linked to their position on the overview.

After completion of the full validation, the final full model was subsequently interpreted to explain the differences in activity of the compounds on the individual mutants. This is the model we used to perform the experimental validation and not one of the LOSO models. Firstly we focused on the sequence side of the model (Table 1 and for a multiple sequence alignment see Figure S6). By correlating model predictions of all compounds on each mutant sequence, where all amino acids were replaced in turn by their wild type counterpart on that particular position, an overview of the variation present at all individual residues was created (Figure 7A). The full model explains the lowered activity (pEC_50_) on mutants mainly by mutations at residues 100, 101, 103, 162, 179, 181, 188, 227, 234 and slightly by residue 138 on the ‘b’ chain. Interestingly the model interprets mutations at positions 89, 102, 118, 190, 203, 207, 210, 215, 219, 245 to actually slightly increase compound activity. Residues 106, 169 and 214 have little influence in this model and residue 211 does not seem to contribute at all (Figure S7 shows the variation in pEC_50_ caused by individual mutations).

The main contributor to lowered affinity appears to be residue 179 rather than residue 181, while the latter is known to lead to NNRTI cross resistance [60]. Supporting this finding, it is known that clinically used Etravirine, a compound similar to but not included in our data set, is also sensitive to mutations at position 179 (V179D, V179F, and V179T [60]). Secondly, the influence of mutations at position 101 differs widely indicated by the high standard deviation. This is caused mainly by the K101P mutant, which causes a large decrease in pEC_50_ for some compounds and very little for others, depending on the chemistry (see below). The K101E mutant overall has little influence. This interpretation is in line with the results from the LOSO experiments mentioned above where both residues 101 and 179 were identified as having a high impact on model reliability.

### Model based interpretation of ligand substructures

Similar to the mutation interpretation, the full model was interpreted to elucidate the average contribution of individual compound substructures to changes in activity (Figure 7B). Here the substructure that was being investigated was replaced by a single carbon atom in all compounds and the subsequent model predictions were compared to the model predictions for the original compounds. Figure 7B shows the contribution of the substructures after a selection was made to only use substructures that occur in more than one compound in order to lower a bias of a continuously active compound (resulting in 1068 of 2546 substructures). In the figure some examples of substructures that improve activity and substructures that decrease activity are shown. For a full table with the top 15 best and top 15 worst substructures, please see Table S1 and Table S2. Figure 6 (compounds **5,6**) shows two examples of compounds that contain a substructure, which has been highlighted, that the model predicts to lead to a good activity and two examples of compounds (**7,8**) that contain a substructure that the model predicts to have a negative effect on activity. The effect is expressed as an average increase or decrease of all compounds containing that substructure and their activities on all sequences. Compound **5** contains the 17^th^ best substructure, leading to an average increase in pEC_50_ of 0.14, and compound **6** contains the 30^th^ best substructure, leading to an average increase in pEC_50_ of 0.12. Therefore these substructures constitute chemistry that is optimally contained in the compound. Compound **7** contains the 3^rd^ worst substructure, leading to an average decrease in pEC_50_ of 0.23, and compound **8** contains the 4^th^ worst substructure, leading to average decrease in pEC_50_ of 0.19. These two substructures constitute chemistry that is rather avoided in possible drug candidates.

### Application of PCM in preclinical drug research

We have shown that PCM can be applied in a preclinical setting to predict the resistance profile of compounds. In addition we can interpret our final full model and identify favorable and unfavorable substructures, providing insights that can be used in compound design. For this data set we can conclude that the mutations in the protein sequences have a larger impact on pEC_50_ values than the compound substructures. However, we have also shown that the substructures still possess a significant influence, as PCM was the only technique that was able to combine target and substructures information.

Combining all our results from prospective experimental validation, the LOSO and the model interpretation, we feel confident that our model can be used to estimate the activity of previously untested compound – sequence pairs, with the main limitation (which can be quantified) being the similarity of the target protein. This opens the door for models that are able to predict the changes in activity of different compounds on clinical isolates obtained from patients [13]. PCM can thereby serve as a modeling tool to predict the activity for untested compound – isolate pairs before any assay measurement is performed, providing a quick guidance to medicinal chemists in the development of drugs based on their expected resistance profile. An example of this application is given in Tables 3 and 4, listing which inhibitors show best activity against a particular sequence and for which inhibitors resistance would be expected. (Table S4 lists a total of 57 compounds and their activity on the 14 sequences) Likewise, our method also aids in the development of drugs that have a broad inhibition profile. Noteworthy is that eight of the 28 specific compound - sequence predictions are untested compound – sequence pairs, which underlines the value of PCM to extrapolate to untested drug – sequence pairings, a feature not possible to achieve in conventional (single-target based) bioactivity modeling.

**Table 3:** Best performing compounds (per sequence and overall).

Sequence	Compound with highest pEC_50_	Activity (pEC_50_)	Full Model (pEC_50_)	Difference (Activity and Model)
All	326	8.39 (±0.61)	8.53 (±0.73)	0.14
1	365	9.16	9.55	0.39
2	221	8.19	8.38	0.19
3	79	8.71	8.81	0.10
4	321	8.83	8.79	0.04
5	321	9.12	8.73	0.39
6	221	8.01	7.93	0.08
7	364	untested	7.50	n/a
8	221	untested	8.42	n/a
9	365	untested	9.43	n/a
10	326	untested	9.23	n/a
11	151	9.05	8.86	0.19
12	321	untested	9.29	n/a
13	100	9.06	8.87	0.19
14	79	9.51	9.62	0.11
			Average	0.18

Overview of the compounds with the highest pEC_50_ as obtained from the model. Shown are the pEC_50_ values differentiated over all sequences (all) or per sequence. Also shown is the standard deviation of the distribution over all sequences used to calculate this mean value. It should be noted that compound 326 was not tested on sequences 9, 10 and 14, illustrating the importance of extrapolating in bioactivity space.

**Table 4:** Worst performing compounds (per sequence and overall).

Sequence	Compound with Lowest pEC_50_	Activity (pEC_50_)	Full Model (pEC_50_)	Difference (Activity and Model)
All	109	5.85 (±0.54)	5.82 (±0.66)	0.03
1	248	6.09	6.01	0.08
2	109	untested	4.87	n/a
3	422	untested	5.78	n/a
4	84	5.84	5.67	0.17
5	84	5.65	5.54	0.11
6	109	4.60	4.06	0.54
7	439	5.01	5.20	0.19
8	84	4.74	5.20	0.46
9	248	untested	5.96	n/a
10	181	5.82	6.01	0.19
11	181	5.42	5.61	0.19
12	109	5.90	6.09	0.19
13	181	5.11	5.29	0.18
14	181	5.62	5.81	0.19
			Average	0.21

Overview of the compounds with the lowest pEC_50_ as obtained from the model. Shown are the pEC_50_ values differentiated over all sequences (all) or per sequence. Also shown is the standard deviation of the distribution over all sequences used to calculate this mean value. It should be noted that compound 109 was not tested on sequences 2, 7 and 8, illustrating the importance of extrapolating in bioactivity space.

### Conclusions

In this work we have shown how to incorporate personalized data (specific viral mutants) as a tool to select optimal candidates in drug development by using proteochemometrics modeling in combination with a large scale experimental validation of inhibitors of HIV Reverse Transcriptase. While applied here to NNRTIs, PCM is a universally applicable method since all it requires is the sequence of a target of interest and structures of ligands. These two prerequisites are something that is available in any preclinical drug research project. We employed a prospective validation of 317 new experimental data points and a new type of ‘leave one sequence out’ validation (representing the case of a previously untested virus genotype). We were able to predict which compounds are best for a particular HIV RT sequence (with high accuracy in 12 out of the 14 sequences in the data set). We established that distance in biological (target) space is tightly correlated with prediction performance, enabling us to judge where the model likely succeeds, and where it may fail. Hence, in this work, we present a real-world scenario of HIV drug development, make practical steps towards drug design tailored towards specific patients, and we aim to extend this to other target families in the future.

### Supporting Information

Additional figures (Figures S1, S2, S3, S4, S5, S6, S7, S8, S9, S10, S11), four tables (Tables S1, S2, S3, S4), the final model and 57 of the here modeled compounds with biological activity ( are available online. A protocol to be run in pipeline pilot to apply this model and perform PCM is also available online.

## Supporting Information

Figure S1 **Correlation parameters plotted against the average similarity with the training set.** (A) The R_0_^2^ of the different leave-one-sequence-out experiments (LOSO) against the average similarity between that specific sequence and the training set. (B) The RMSE of the different leave-one-sequence-out experiments against the average similarity between that specific sequence and the training set.
(TIF)

Figure S2 **The distribution of the correlation parameters when validating individual compound predictions using the individual LOSO models.** (A) Distribution of the RMSE and the R_0_^2^ of the individual compound predictions using the LOSO models. The compounds have been ranked by increasing RMSE in (A), the corresponding R_0_^2^ is shown in (B). Please note that the number on the x-axis is not the name of the compound, it is merely a serial number. Likewise the compounds have been ranked by increasing R_0_^2^ (C) the corresponding RMSE is shown in (D).
(TIF)

Figure S3 **Two compounds that were predicted accurately using the LOSO models.** (A) Prediction of the activity (pEC_50_ value) of compound 1 on the different sequences using the LOSO models (RMSE of 0.22 log units, R_0_^2^ of 0.96). (B) Prediction of the activity (pEC_50_ value) of compound 2 on the different sequences using the LOSO models (RMSE of 0.26 log units, R_0_^2^ of 0.89).
(TIF)

Figure S4 **Two compounds that were predicted inaccurately using the LOSO models.** (A) Prediction of the activity (pEC_50_ value) of compound 3 on the different sequences using the LOSO models (RMSE of 1.12 log units, R_0_^2^ of −0.10). (B) Prediction of the activity (pEC_50_ value) of compound 4 on the different sequences using the LOSO models (RMSE of 0.61 log units, R_0_^2^ of −3.66).
(TIF)

Figure S5 **Sequences present in the dataset clustered to similarity based on the protein descriptor.**
(TIF)

Figure S6 **Multiple sequence alignment of the used mutants.** Outside the shown alignment the 14 sequences were equal; therefore these residues were omitted from the PCM model.
(TIF)

Figure S7 **Average contribution to pEC_50_ according to the full model of all present mutants.** The standard deviation was determined over all calculated pEC_50_ changes.
(TIF)

Figure S8 **Overview of the pEC_50_ values of all compound – sequence pairs.**
(TIF)

Figure S9 **PCM and QSAR learning curves.** As training is performed on an increasing part of the data set, validation is performed on a decreasing part of the data set. The PCM models are shown by solid lines and the QSAR by dashed lines. The validation parameters were calculated per sequence, using a single PCM model for all sequences, as well as for comparison and dedicated QSAR models for each individual sequence. The error bars indicate the standard deviation over the R_0_^2^ and RMSE values of the validation on the different sequences. The PCM single models outperform the dedicated QSAR models in each case both measured by the R_0_^2^ and the RMSE.
(TIF)

Figure S10 **The maximal distance of the compound from the training set plotted to the prediction error.** The plot shows that the distance relates to the prediction error as compounds closer than 0.98 are predicted better than compounds further away than 0.98 from the training set.
(TIF)

Figure S11 **Y-scrambling plot.** In order to rule out chance correlations 100-fold Y-scrambling was performed. After scrambling the pEC_50_ values, these 100 data sets were in each case divided into a training set consisting of 80% of the total set and a test set consisting of 20%. The models built on permutated data cannot be validated with a Q^2^, R^2^ and R_0_^2^ of approximately 0. After training and validation, Q^2^, R^2^ of the training and R_0_^2^ of the validation were plotted against the similarity of the scrambled dataset with the original training set. This correlation was defined as the % of compounds that had a pEC_50_ value within 0.3 log units of its true value. A simple linear regression was subsequently performed for Q^2^, R^2^ and R_0_^2^. The regression lines for Q^2^, R^2^ of the training and R_0_^2^ of the validation crossed the y-axis at −0.12, −0.10 and −0.16 respectively. We conclude that it is highly unlikely that our model was created based on chance correlations between the different descriptors. Therefore we are modeling an actual correlation between the pEC_50_ values on the one hand and the compounds and proteins on the other hand.
(TIF)

Table S1 **The top 15 substructures contributing the most to binding on all sequences.** Mean indicates the mean increase in pEC_50_ by the presence of that particular substructure, StdDev represents the standard deviation of the distribution of all changes in pEC_50_ correlated with the presence of that substructure and N represents the amount of predicted changes within this distribution. Binvalue is the identifier for that particular substructure (FCFP_6 format) and the properties Variance, Skew and Kurtosis have also been calculated over the distribution to determine the distribution pattern of the changes caused by that substructure.
(DOC)

Table S2 **The top 15 substructures leading on average to a lower pEC_50_ on all sequences.** Mean indicates the mean decrease in pEC_50_ by the presence of that particular substructure, StdDev represents the standard deviation of the distribution of all changes in pEC_50_ correlated with the presence of that substructure and N represents the amount of predicted changes within this distribution. Binvalue is the identifier for that particular substructure (FCFP_6 format) and the properties Variance, Skew and Kurtosis have also been calculated over the distribution to determine the distribution pattern of the changes caused by that substructure.
(DOC)

Table S3 **All AAindices used with their reference on the AAindex website.** This table lists all indices we used to create a unique hashed value per amino acid.
(DOC)

Table S4 **The complete SAR of 57 of the modeled compounds.**
(DOC)

Archive S1 **Model and sample data set.**
(RAR)
