# Supplementary figures and images for: Which Compound to Select in Lead Optimization? Prospectively Validated Proteochemometric Models Guide Preclinical Development
Source: PLoS One. 2011 Nov 23;6(11):e27518. doi: 10.1371/journal.pone.0027518 (PMC3223189; doi:10.1371/journal.pone.0027518)

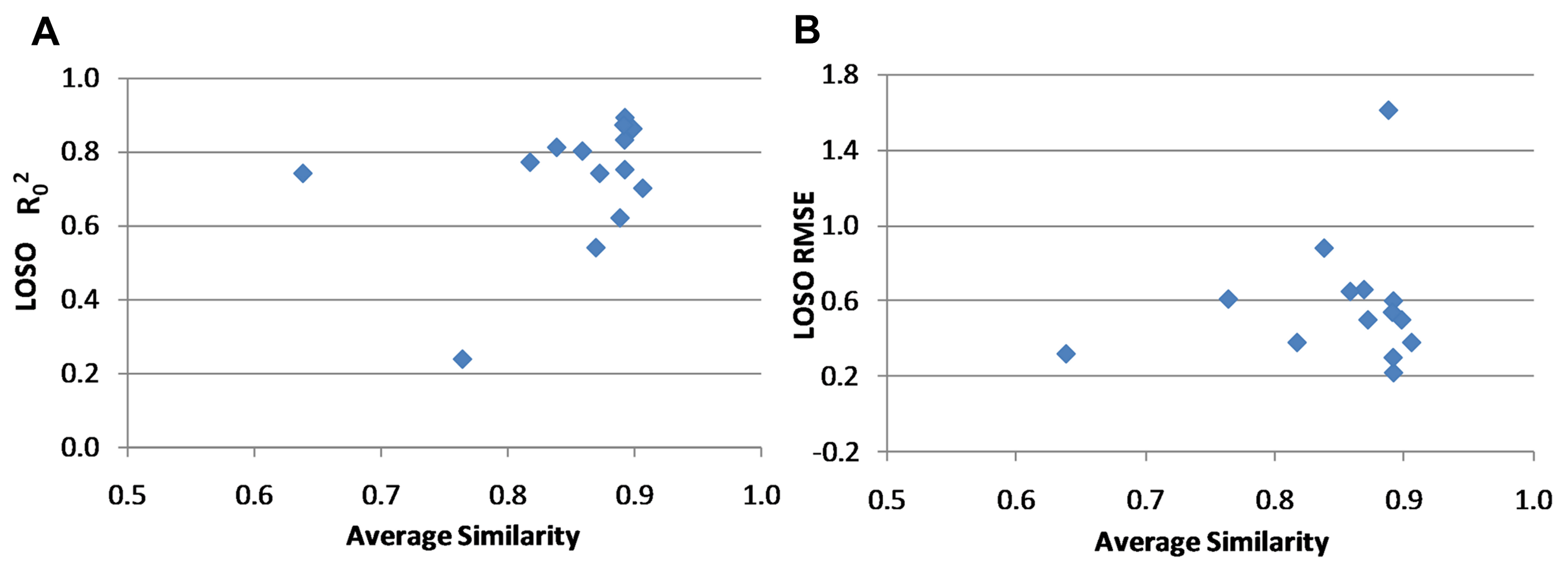

Supplement: Figure S1 — Correlation parameters plotted against the average similarity with the training set. (A) The R0 2 of the different leave-one-sequence-out experiments (LOSO) against the average similarity between that specific sequence and the training set. (B) The RMSE of the different leave-one-sequence-out experiments against the average similarity between that specific sequence and the training set. (TIF) [file pone.0027518.s001.tif]

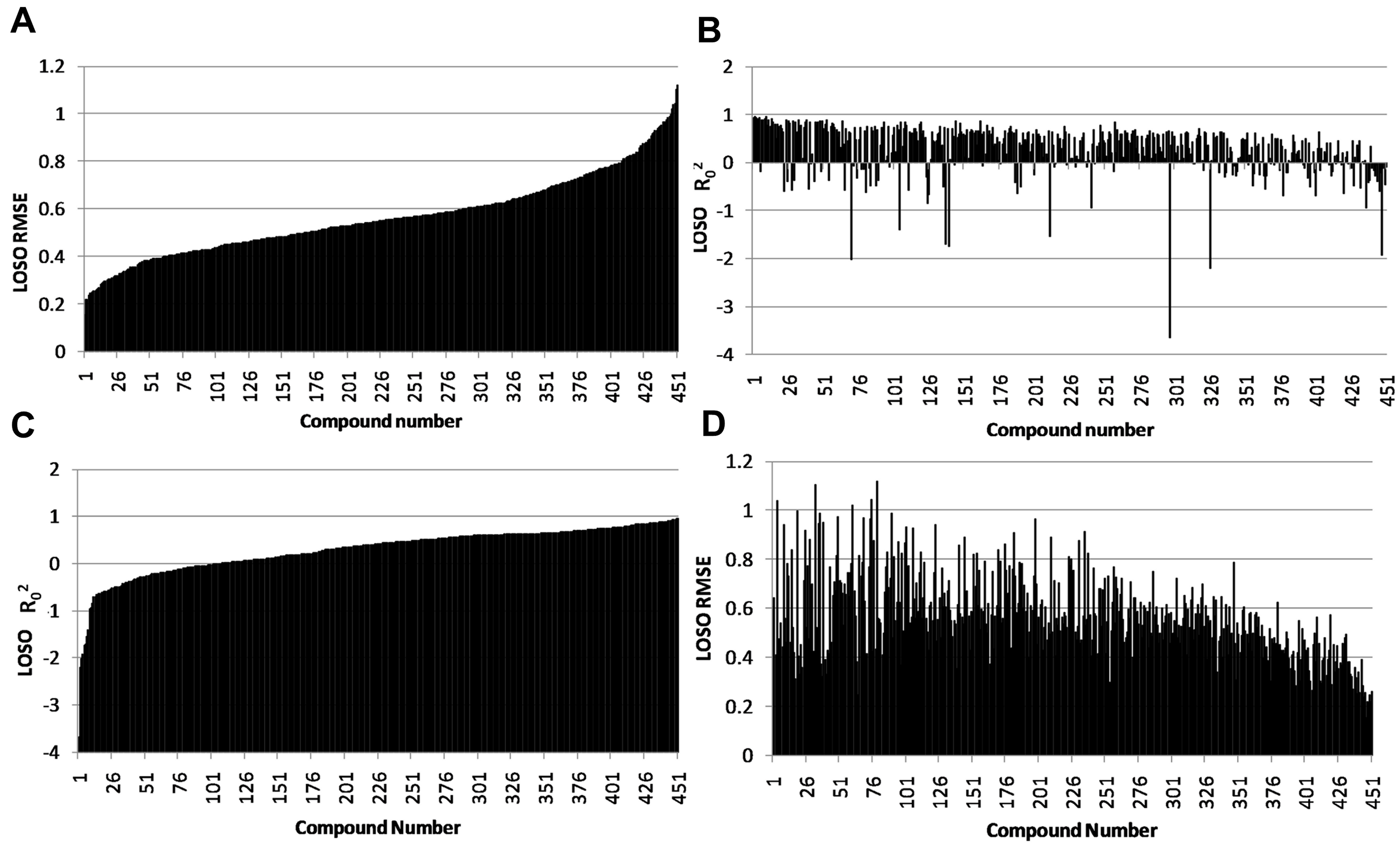

Supplement: Figure S2 — The distribution of the correlation parameters when validating individual compound predictions using the individual LOSO models. (A) Distribution of the RMSE and the R0 2 of the individual compound predictions using the LOSO models. The compounds have been ranked by increasing RMSE in (A), the corresponding R0 2 is shown in (B). Please note that the number on the x-axis is not the name of the compound, it is merely a serial number. Likewise the compounds have been ranked by increasing R0 2 (C) the corresponding RMSE is shown in (D). (TIF) [file pone.0027518.s002.tif]

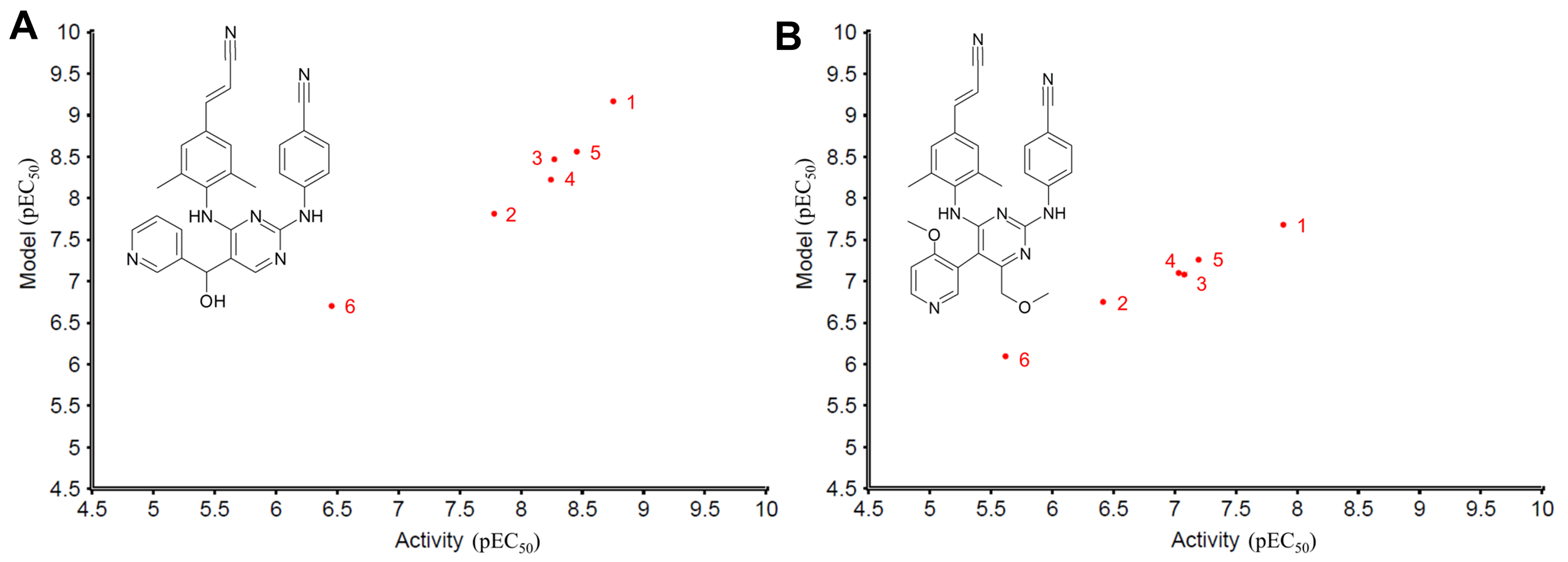

Supplement: Figure S3 — Two compounds that were predicted accurately using the LOSO models. (A) Prediction of the activity (pEC50 value) of compound 1 on the different sequences using the LOSO models (RMSE of 0.22 log units, R0 2 of 0.96). (B) Prediction of the activity (pEC50 value) of compound 2 on the different sequences using the LOSO models (RMSE of 0.26 log units, R0 2 of 0.89). (TIF) [file pone.0027518.s003.tif]

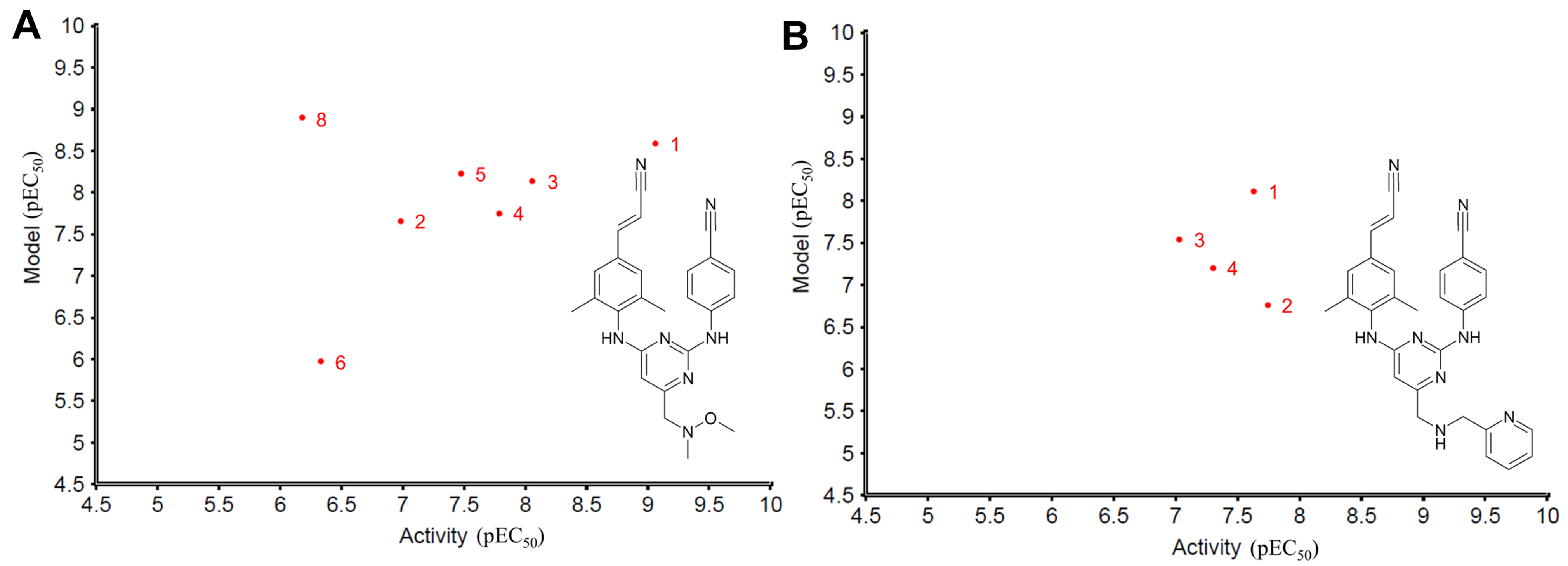

Supplement: Figure S4 — Two compounds that were predicted inaccurately using the LOSO models. (A) Prediction of the activity (pEC50 value) of compound 3 on the different sequences using the LOSO models (RMSE of 1.12 log units, R0 2 of −0.10). (B) Prediction of the activity (pEC50 value) of compound 4 on the different sequences using the LOSO models (RMSE of 0.61 log units, R0 2 of −3.66). (TIF) [file pone.0027518.s004.tif]

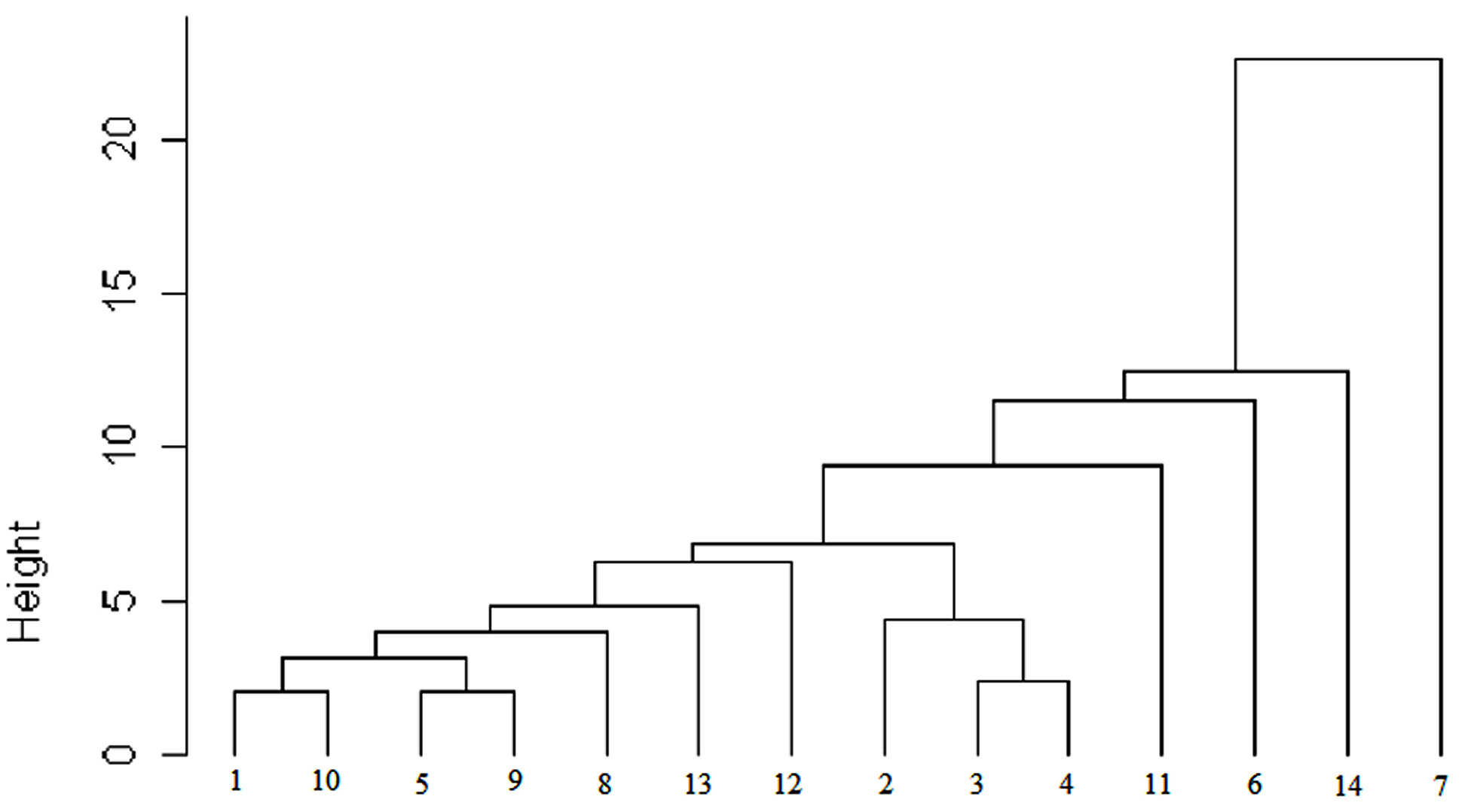

Supplement: Figure S5 — Sequences present in the dataset clustered to similarity based on the protein descriptor. (TIF) [file pone.0027518.s005.tif]

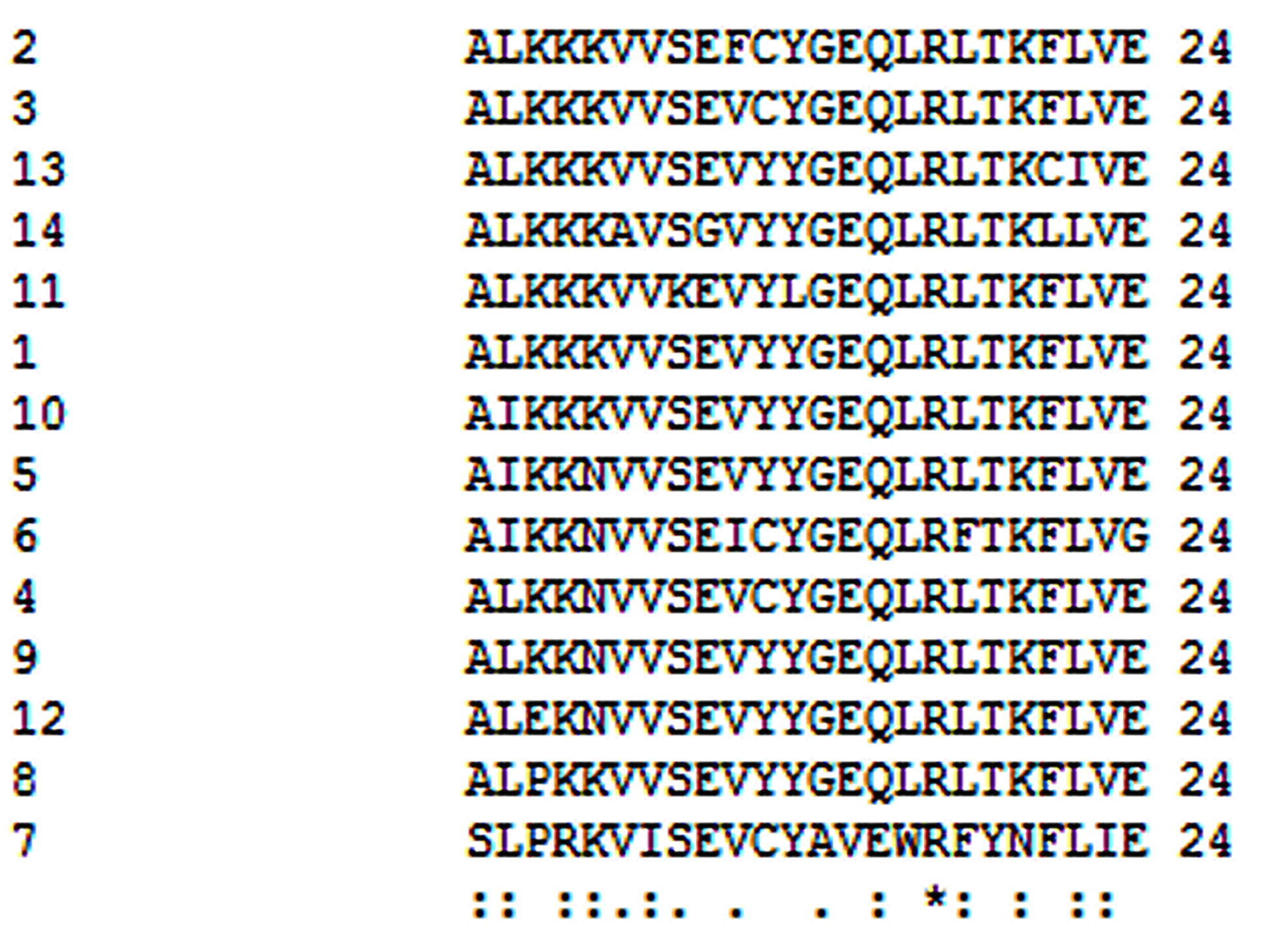

Supplement: Figure S6 — Multiple sequence alignment of the used mutants. Outside the shown alignment the 14 sequences were equal; therefore these residues were omitted from the PCM model. (TIF) [file pone.0027518.s006.tif]

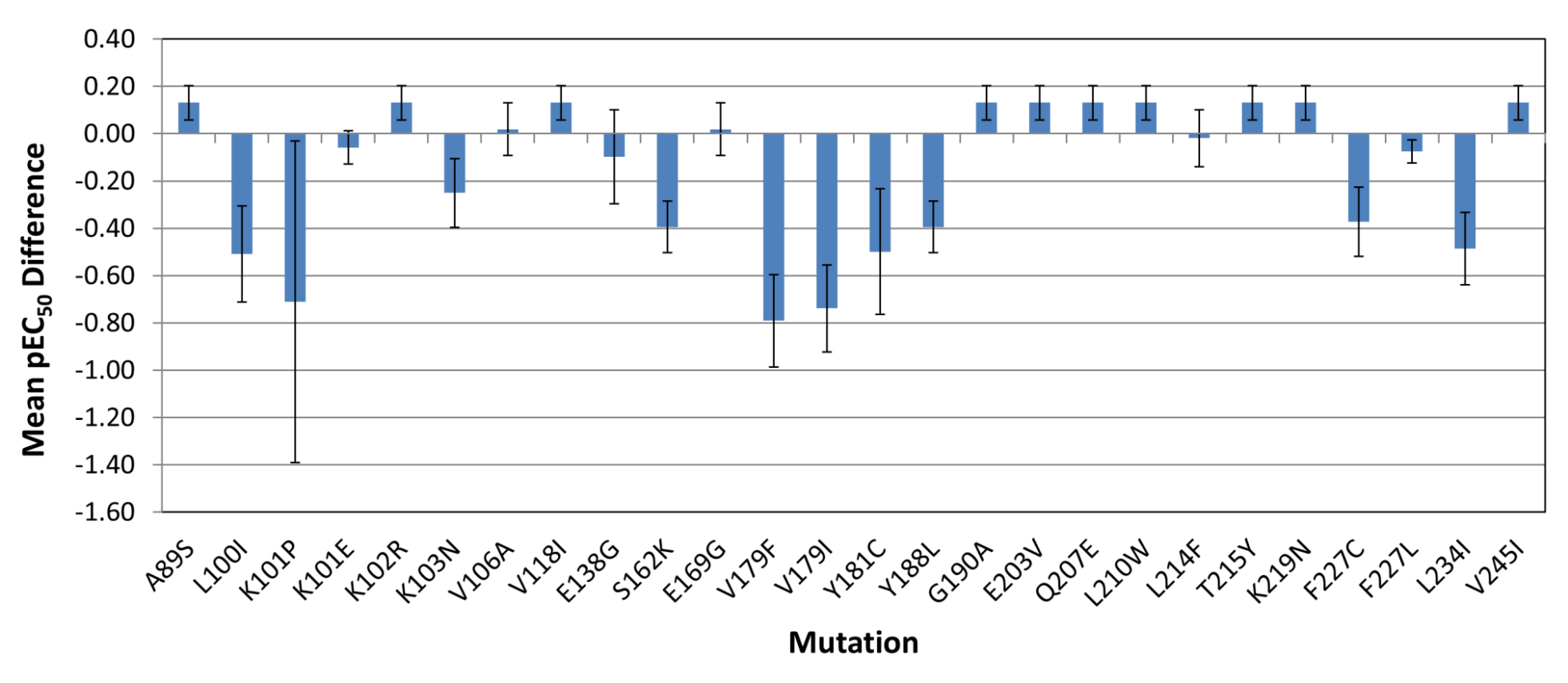

Supplement: Figure S7 — Average contribution to pEC50 according to the full model of all present mutants. The standard deviation was determined over all calculated pEC50 changes. (TIF) [file pone.0027518.s007.tif]

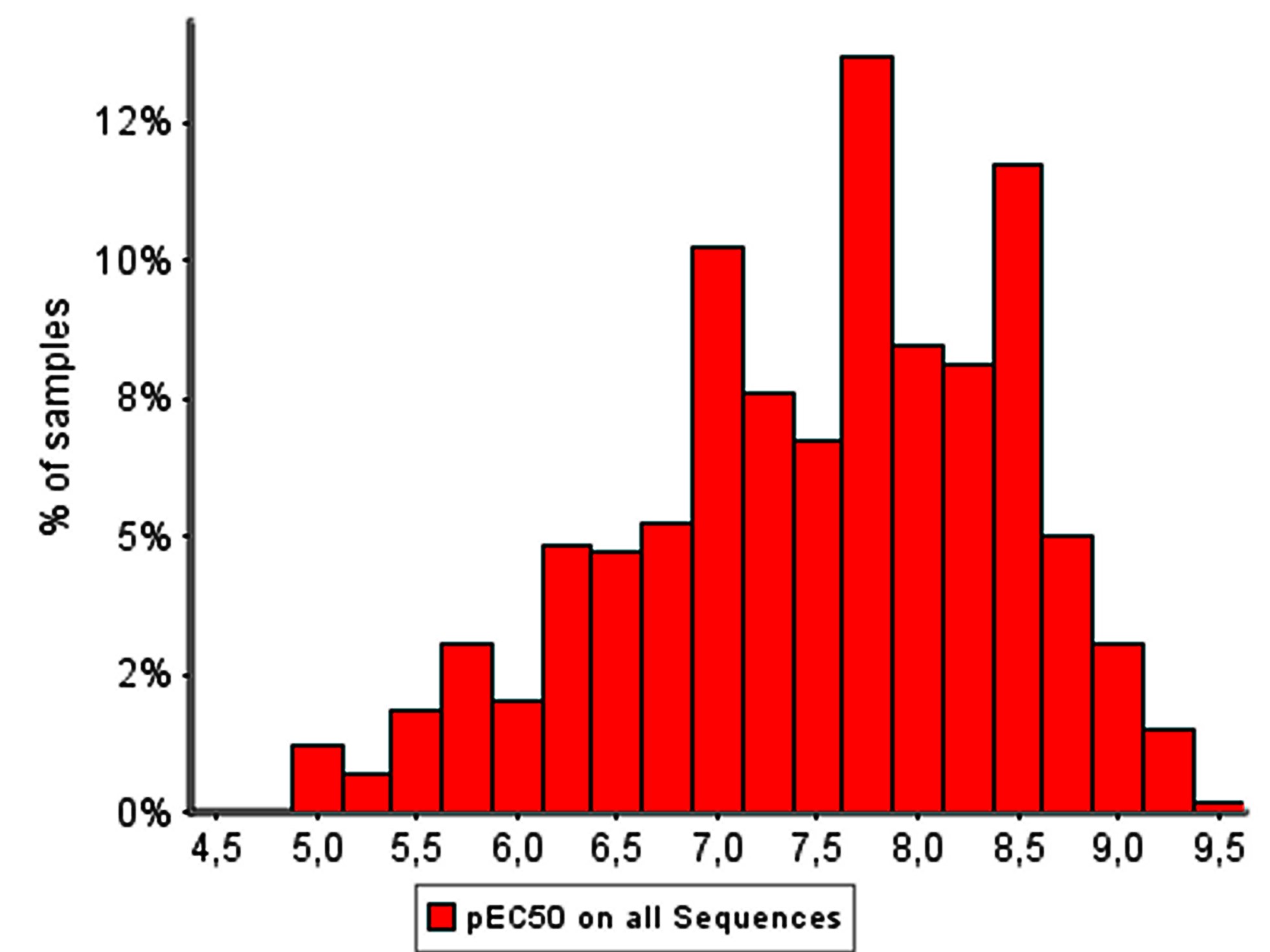

Supplement: Figure S8 — Overview of the pEC50 values of all compound – sequence pairs. (TIF) [file pone.0027518.s008.tif]

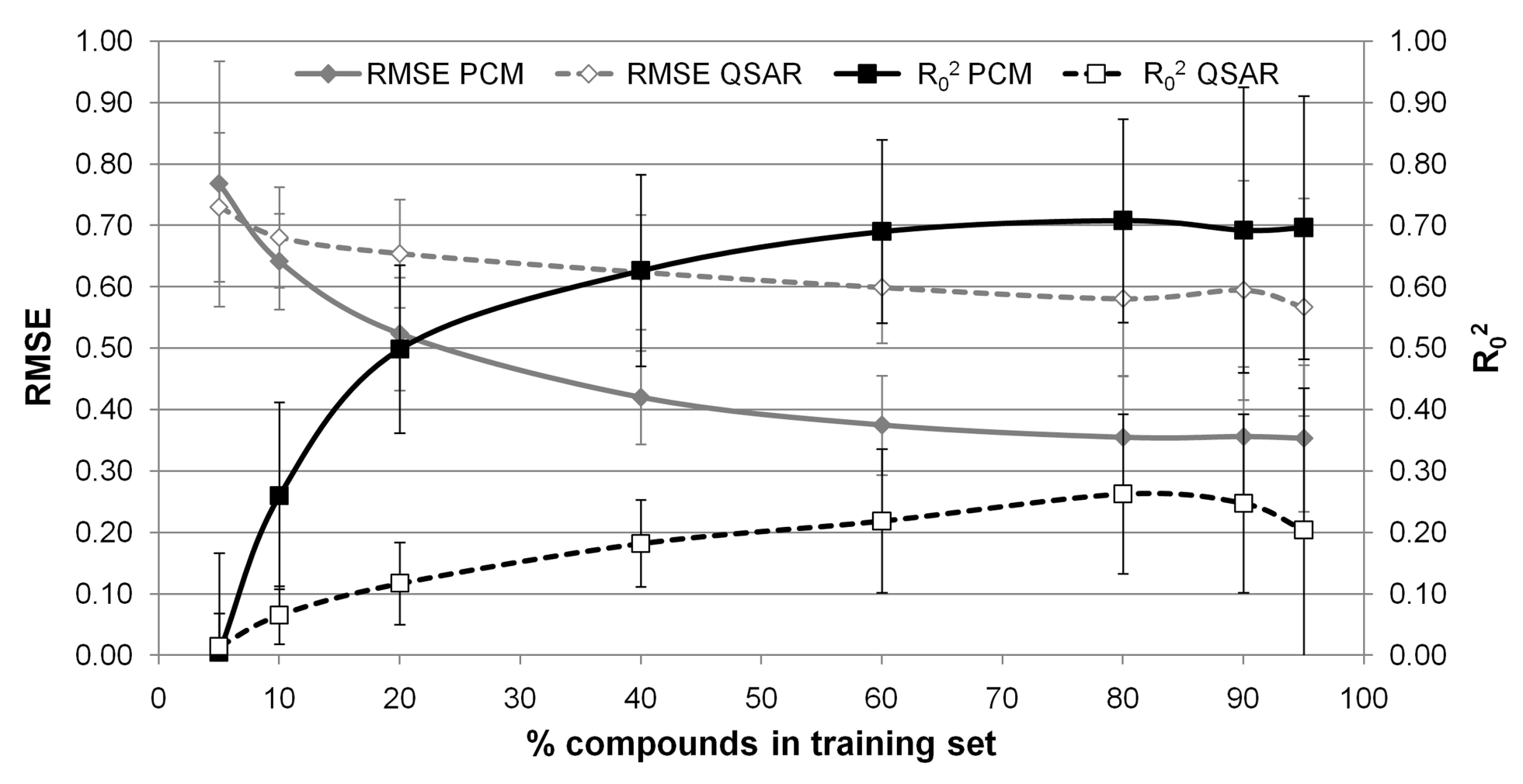

Supplement: Figure S9 — PCM and QSAR learning curves. As training is performed on an increasing part of the data set, validation is performed on a decreasing part of the data set. The PCM models are shown by solid lines and the QSAR by dashed lines. The validation parameters were calculated per sequence, using a single PCM model for all sequences, as well as for comparison and dedicated QSAR models for each individual sequence. The error bars indicate the standard deviation over the R0 2 and RMSE values of the validation on the different sequences. The PCM single models outperform the dedicated QSAR models in each case both measured by the R0 2 and the RMSE. (TIF) [file pone.0027518.s009.tif]

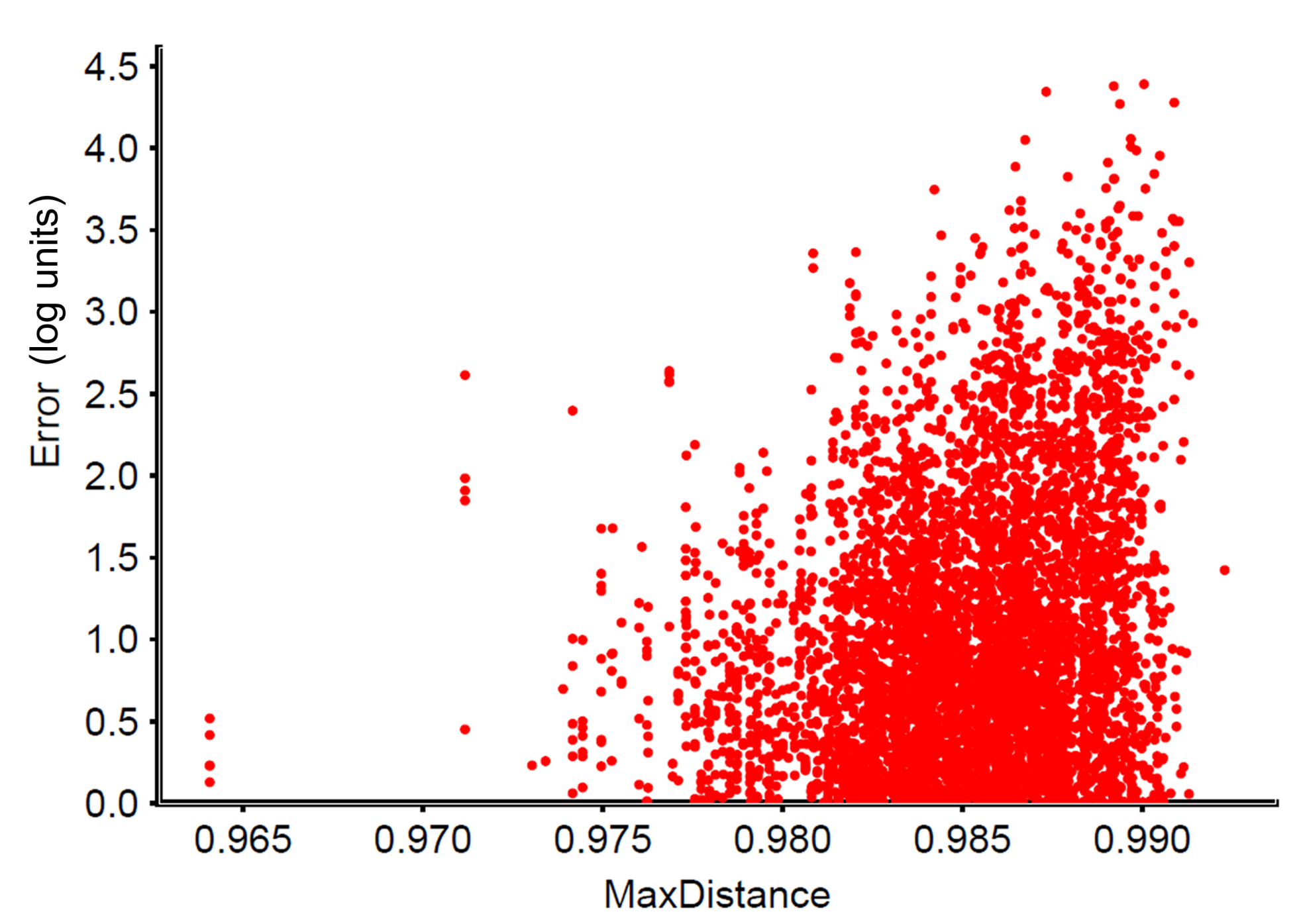

Supplement: Figure S10 — The maximal distance of the compound from the training set plotted to the prediction error. The plot shows that the distance relates to the prediction error as compounds closer than 0.98 are predicted better than compounds further away than 0.98 from the training set. (TIF) [file pone.0027518.s010.tif]

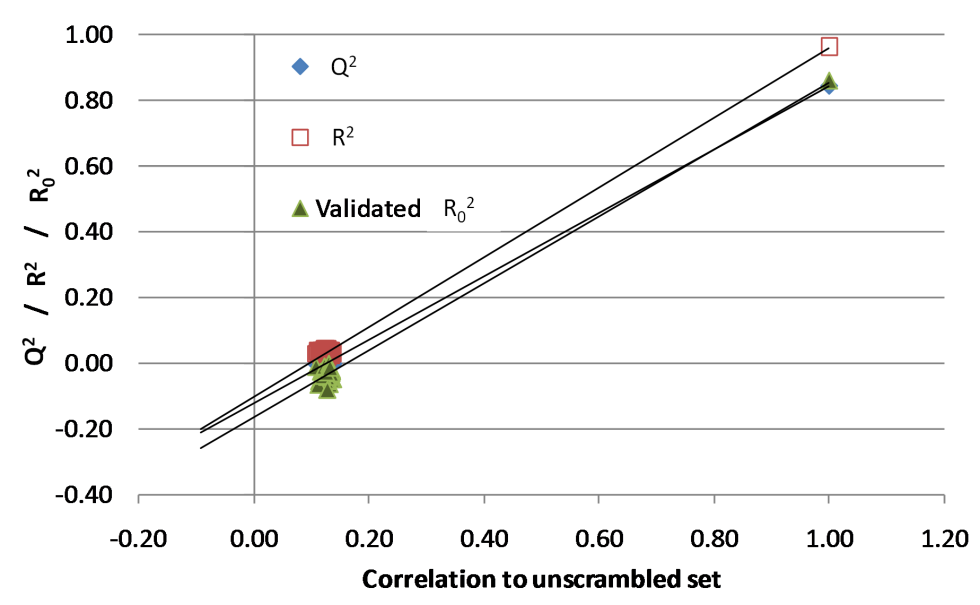

Supplement: Figure S11 — Y-scrambling plot. In order to rule out chance correlations 100-fold Y-scrambling was performed. After scrambling the pEC50 values, these 100 data sets were in each case divided into a training set consisting of 80% of the total set and a test set consisting of 20%. The models built on permutated data cannot be validated with a Q2, R2 and R0 2 of approximately 0. After training and validation, Q2, R2 of the training and R0 2 of the validation were plotted against the similarity of the scrambled dataset with the original training set. This correlation was defined as the % of compounds that had a pEC50 value within 0.3 log units of its true value. A simple linear regression was subsequently performed for Q2, R2 and R0 2. The regression lines for Q2, R2 of the training and R0 2 of the validation crossed the y-axis at −0.12, −0.10 and −0.16 respectively. We conclude that it is highly unlikely that our model was created based on chance correlations between the different descriptors. Therefore we are modeling an actual correlation between the pEC50 values on the one hand and the compounds and proteins on the other hand. (TIF) [file pone.0027518.s011.tif]
