# Supplementary material for: Which Compound to Select in Lead Optimization? Prospectively Validated Proteochemometric Models Guide Preclinical Development
Source: PLoS One. 2011 Nov 23;6(11):e27518. doi: 10.1371/journal.pone.0027518 (PMC3223189; doi:10.1371/journal.pone.0027518)
Supplement: Table S1 — The top 15 substructures contributing the most to binding on all sequences. Mean indicates the mean increase in pEC50 by the presence of that particular substructure, StdDev represents the standard deviation of the distribution of all changes in pEC50 correlated with the presence of that substructure and N represents the amount of predicted changes within this distribution. Binvalue is the identifier for that particular substructure (FCFP_6 format) and the properties Variance, Skew and Kurtosis have also been calculated over the distribution to determine the distribution pattern of the changes caused by that substructure. (DOC) [file pone.0027518.s012.doc]

Table S1. 15 substructures contributing most to binding on all sequences.

| Substructure | Mean | StdDev | N | Binvalue | Variance | Skew | Kurtosis |
| --- | --- | --- | --- | --- | --- | --- | --- |
|  | 1.03 | 1.17 | 28 | -1742025611 | 1.38 | 0.16 | -1.90 |
|  | 0.60 | 0.84 | 56 | 1620855811 | 0.70 | 1.15 | -0.40 |
|  | 0.36 | 0.19 | 6314 | 16 | 0.04 | -0.61 | -0.08 |
|  | 0.31 | 0.31 | 42 | 1830122116 | 0.10 | 1.02 | -0.70 |
|  | 0.27 | 0.32 | 28 | 1624151150 | 0.10 | 0.31 | -1.75 |
|  | 0.27 | 0.12 | 28 | -1746457593 | 0.01 | -1.10 | 1.76 |
|  | 0.26 | 0.17 | 28 | 1021957095 | 0.03 | -0.05 | -1.12 |
|  | 0.22 | 0.16 | 56 | -1968900341 | 0.02 | -0.40 | -1.14 |
|  | 0.22 | 0.29 | 28 | -1171019670 | 0.09 | 0.20 | -1.85 |
|  | 0.22 | 0.15 | 42 | 1588141279 | 0.02 | 0.73 | -0.78 |
|  | 0.22 | 0.17 | 42 | 803841221 | 0.03 | -0.02 | -1.55 |
|  | 0.20 | 0.22 | 42 | -1697717939 | 0.05 | 0.55 | -1.22 |
|  | 0.20 | 0.28 | 84 | -1156087477 | 0.08 | 1.36 | 1.09 |
|  | 0.20 | 0.17 | 28 | 1382379231 | 0.03 | -2.15 | 6.49 |
|  | 0.19 | 0.13 | 42 | 1395545170 | 0.02 | 0.15 | -1.09 |

The top 15 substructures contributing the most to binding on all sequences. Mean indicates the mean increase in pEC_50_ by the presence of that particular substructure, StdDev represents the standard deviation of the distribution of all changes in pEC_50_ correlated with the presence of that substructure and N represents the amount of predicted changes within this distribution. Binvalue is the identifier for that particular substructure (FCFP_6 format) and the properties Variance, Skew and Kurtosis have also been calculated over the distribution to determine the distribution pattern of the changes caused by that substructure.
