# Supplementary material for: Which Compound to Select in Lead Optimization? Prospectively Validated Proteochemometric Models Guide Preclinical Development
Source: PLoS One. 2011 Nov 23;6(11):e27518. doi: 10.1371/journal.pone.0027518 (PMC3223189; doi:10.1371/journal.pone.0027518)
Supplement: Table S3 — All AAindices used with their reference on the AAindex website. This table lists all indices we used to create a unique hashed value per amino acid. (DOC) [file pone.0027518.s014.doc]

Table S3**.** Overview of the AAindices used to create a unique hashed value per amino acid

| Number | AAindex Identifier | Descriptor |
| --- | --- | --- |
| 1 | ARGP820103 | Membrane-buried preference parameters |
| 2 | BAEK050101 | Linker index |
| 3 | BHAR880101 | Average flexibility indices |
| 4 | CASG920101 | Hydrophobicity scale from native protein structures |
| 5 | CHAM810101 | Steric parameter |
| 6 | CHAM820101 | Polarizability parameter |
| 7 | CHAM830101 | The Chou-Fasman parameter of the coil conformation |
| 8 | CHAM830107 | A parameter of charge transfer capability |
| 9 | CHAM830108 | A parameter of charge transfer donor capability |
| 10 | CHOP780201 | Normalized frequency of alpha-helix |
| 11 | CHOP780202 | Normalized frequency of beta-sheet |
| 12 | CHOP780203 | Normalized frequency of beta-turn |
| 13 | CIDH920105 | Normalized average hydrophobicity scales |
| 14 | COSI940101 | Electron-ion interaction potential values |
| 15 | FASG760101 | Molecular weight |
| 16 | FAUJ880102 | Smoothed upsilon steric parameter |
| 17 | FAUJ880103 | Normalized van der Waals volume |
| 18 | FAUJ880104 | STERIMOL length of the side chain |
| 19 | FAUJ880105 | STERIMOL minimum width of the side chain |
| 20 | FAUJ880106 | STERIMOL maximum width of the side chain |
| 21 | FAUJ880109 | Number of hydrogen bond donors |
| 22 | FAUJ880110 | Number of full nonbonding orbitals |
| 23 | FAUJ880111 | Positive charge |
| 24 | FAUJ880112 | Negative charge |
| 25 | FAUJ880113 | pK-a(RCOOH) |
| 26 | GRAR740102 | Polarity |
| 27 | JANJ780102 | Percentage of buried residues |
| 28 | JANJ780103 | Percentage of exposed residues |
| 29 | JOND920102 | Relative mutability |
| 30 | JUNJ780101 | Sequence frequency |
| 31 | KLEP840101 | Net charge |
| 32 | KOEP990101 | Alpha-helix propensity derived from designed sequences |
| 33 | KOEP990102 | Beta-sheet propensity derived from designed sequences |
| 34 | KRIW790101 | Side chain interaction parameter |
| 35 | KYTJ820101 | Hydropathy index |
| 36 | LEVM760102 | Distance between C-alpha and centroid of side chain |
| 37 | LEVM760103 | Side chain angle theta(AAR) |
| 38 | LEVM760104 | Side chain torsion angle phi(AAAR) |
| 39 | LEVM760105 | Radius of gyration of side chain |
| 40 | LEVM760106 | van der Waals parameter R0 |
| 41 | LEVM760107 | van der Waals parameter epsilon |
| 42 | MITS020101 | Amphiphilicity index |
| 43 | MONM990201 | Averaged turn propensities in a transmembrane helix |
| 44 | NISK800101 | 8 A contact number |
| 45 | NISK860101 | 14 A contact number |
| 46 | PONP800101 | Surrounding hydrophobicity in folded form |
| 47 | PONP930101 | Hydrophobicity scales |
| 48 | RACS770103 | Side chain orientational preference |
| 49 | RADA880108 | Mean polarity |
| 50 | ROSG850101 | Mean area buried on transfer |
| 51 | ROSG850102 | Mean fractional area loss |
| 52 | ROSM880102 | Side chain hydropathy, corrected for solvation |
| 53 | TAKK010101 | Side-chain contribution to protein stability |
| 54 | VINM940101 | Normalized flexibility parameters (B-values), average |
| 55 | WARP780101 | Average interactions per side chain atom |
| 56 | WOLR810101 | Hydration potential |
| 57 | ZHOH040102 | The relative stability scale extracted from mutation experiments |
| 58 | ZHOH040103 | Buriability |
