# Supplementary material for: Which Compound to Select in Lead Optimization? Prospectively Validated Proteochemometric Models Guide Preclinical Development
Source: PLoS One. 2011 Nov 23;6(11):e27518. doi: 10.1371/journal.pone.0027518 (PMC3223189; doi:10.1371/journal.pone.0027518)
Supplement: Table S4 — The complete SAR of 57 of the modeled compounds. (DOC) [file pone.0027518.s015.doc]

Table S4. 57 of the modeled compounds and their biological activity spectrum on the 14 targets.

| Name | Activity  (pEC50) | Sequence  Number | Model  (pEC50) | Model  Error | Structure |
| --- | --- | --- | --- | --- | --- |
| 1 | 8.75 7.78 8.27 8.24 8.45 6.46 untested untested untested untested untested untested untested untested | 01 02 03 04 05 06 07 08 09 10 11 12 13 14 | 9.04 7.59 8.38 8.33 8.22 6.27 6.65 7.59 8.94 8.63 7.96 8.83 7.76 8.58 | 0.29 0.19 0.11 0.09 0.23 0.19 n/a n/a n/a n/a n/a n/a n/a n/a | 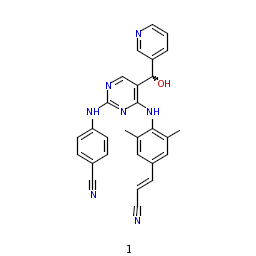 |
| 2 | 7.89 6.41 7.08 7.03 7.20 5.62 untested untested untested untested untested untested untested untested | 01 02 03 04 05 06 07 08 09 10 11 12 13 14 | 7.70 6.59 7.02 6.99 7.14 5.81 6.39 6.35 7.64 7.45 7.10 7.62 6.93 7.62 | 0.19 0.17 0.06 0.04 0.05 0.19 n/a n/a n/a n/a n/a n/a n/a n/a | 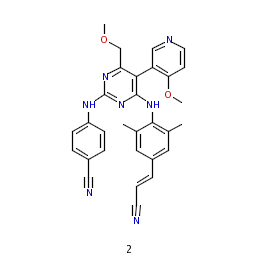 |
| 3 | 9.06 6.98 8.06 7.79 7.48 6.33 untested 6.18 untested untested untested untested untested untested | 01 02 03 04 05 06 07 08 09 10 11 12 13 14 | 8.81 7.16 8.00 7.80 7.67 6.14 6.37 6.59 8.56 8.25 7.76 8.69 7.78 8.71 | 0.25 0.17 0.06 0.02 0.19 0.19 n/a 0.41 n/a n/a n/a n/a n/a n/a | 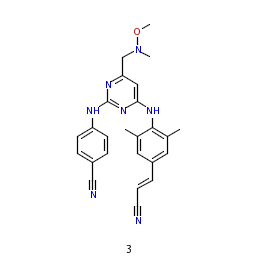 |
| 4 | 7.63 7.75 7.03 7.30 untested 5.75 untested untested untested untested untested untested untested untested | 01 02 03 04 05 06 07 08 09 10 11 12 13 14 | 7.82 6.73 7.31 7.40 7.15 5.41 6.65 6.47 7.87 7.40 7.16 7.79 7.15 7.87 | 0.19 1.01 0.28 0.09 n/a 0.34 n/a n/a n/a n/a n/a n/a n/a n/a | 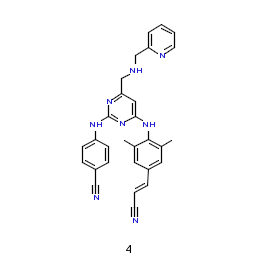 |
| 5 | 8.82 untested 7.25 6.84 6.50 4.91 untested untested 8.43 7.30 7.09 8.24 7.12 7.70 | 01 02 03 04 05 06 07 08 09 10 11 12 13 14 | 8.06 6.26 7.20 7.09 7.01 5.45 6.69 6.54 7.90 7.48 7.06 7.86 6.93 7.89 | 0.76 n/a 0.05 0.25 0.51 0.54 n/a n/a 0.52 0.19 0.02 0.38 0.19 0.19 | 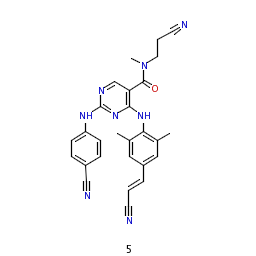 |
| 6 | 7.02 5.67 6.37 6.22 6.28 untested untested untested untested untested untested untested untested untested | 01 02 03 04 05 06 07 08 09 10 11 12 13 14 | 7.20 5.86 6.48 6.31 6.47 5.50 6.19 5.80 6.99 6.95 6.68 6.97 6.58 7.20 | 0.18 0.19 0.11 0.09 0.19 n/a n/a n/a n/a n/a n/a n/a n/a n/a | 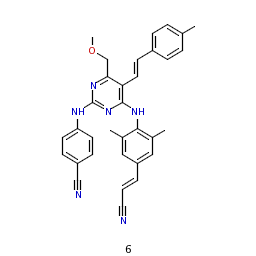 |
| 7 | 8.76 untested 6.98 6.40 5.89 4.95 untested untested 8.31 6.78 6.52 8.21 6.88 untested | 01 02 03 04 05 06 07 08 09 10 11 12 13 14 | 8.02 6.24 7.15 7.17 6.99 5.14 6.91 6.66 8.01 7.31 6.87 7.94 7.07 8.23 | 0.74 n/a 0.16 0.77 1.10 0.19 n/a n/a 0.30 0.54 0.35 0.27 0.18 n/a | 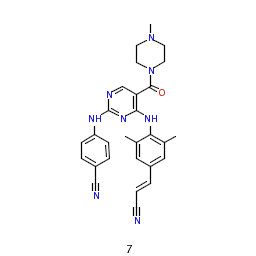 |
| 8 | 8.45 untested 7.72 7.50 7.57 6.35 untested untested 8.21 8.34 7.50 7.78 7.24 untested | 01 02 03 04 05 06 07 08 09 10 11 12 13 14 | 8.54 6.81 7.79 7.68 7.63 6.16 6.54 7.05 8.38 8.12 7.69 8.29 7.30 8.43 | 0.09 n/a 0.07 0.19 0.05 0.19 n/a n/a 0.17 0.22 0.19 0.51 0.06 n/a | 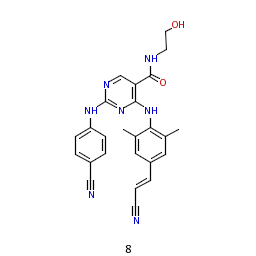 |
| 9 | 8.08 5.58 7.06 7.00 6.73 untested untested 5.59 8.35 7.23 6.52 untested 6.80 7.28 | 01 02 03 04 05 06 07 08 09 10 11 12 13 14 | 7.81 6.62 7.25 7.14 6.91 5.40 6.28 6.64 7.66 7.29 6.71 7.64 6.99 7.47 | 0.27 1.04 0.19 0.13 0.19 n/a n/a 1.05 0.69 0.05 0.19 n/a 0.19 0.19 | 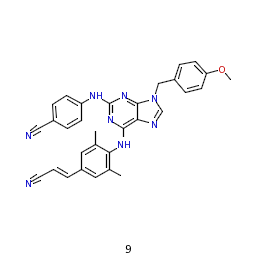 |
| 10 | 8.50 7.20 7.94 8.07 8.09 6.40 untested 7.20 8.51 8.53 7.94 8.17 7.92 8.20 | 01 02 03 04 05 06 07 08 09 10 11 12 13 14 | 8.57 7.14 8.02 7.94 7.97 6.21 6.32 7.39 8.43 8.35 7.75 8.36 7.99 8.39 | 0.06 0.06 0.09 0.13 0.12 0.19 n/a 0.19 0.07 0.19 0.19 0.19 0.08 0.19 | 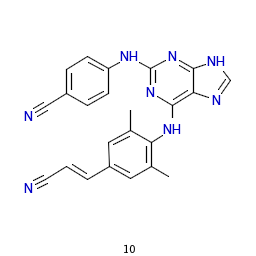 |
| 11 | 6.65 untested 5.71 5.49 5.65 4.60 untested untested 6.38 6.37 5.66 5.90 5.65 6.31 | 01 02 03 04 05 06 07 08 09 10 11 12 13 14 | 6.46 4.87 5.85 5.68 5.81 4.06 6.55 5.35 6.25 6.34 5.84 6.09 5.84 6.50 | 0.19 n/a 0.13 0.19 0.16 0.55 n/a n/a 0.13 0.03 0.19 0.19 0.19 0.19 | 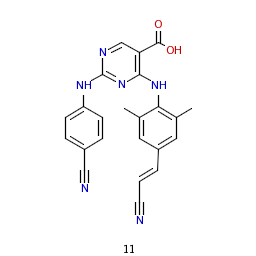 |
| 12 | 8.43 untested 6.97 6.91 6.57 4.86 untested untested 7.68 untested 6.72 7.83 6.28 7.08 | 01 02 03 04 05 06 07 08 09 10 11 12 13 14 | 7.81 5.95 6.88 6.79 6.76 5.37 6.61 6.36 7.68 7.21 6.77 7.64 6.47 7.27 | 0.62 n/a 0.09 0.12 0.19 0.50 n/a n/a 0.00 n/a 0.06 0.19 0.19 0.19 | 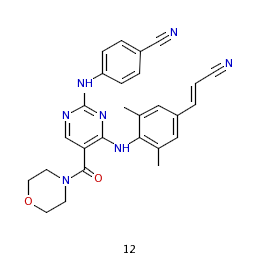 |
| 13 | 9.17 7.52 8.67 9.01 8.79 6.04 untested untested 9.23 9.32 8.70 9.08 8.70 untested | 01 02 03 04 05 06 07 08 09 10 11 12 13 14 | 9.39 7.70 8.75 8.62 8.62 6.23 6.17 7.95 9.21 9.05 8.51 9.17 8.51 9.02 | 0.22 0.19 0.08 0.40 0.18 0.19 n/a n/a 0.03 0.27 0.19 0.09 0.19 n/a | 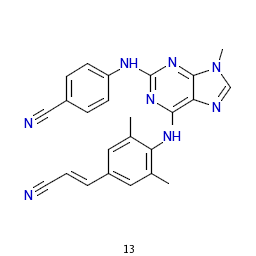 |
| 14 | 8.46 6.68 7.78 7.65 7.15 6.74 7.02 6.99 8.51 7.91 7.63 8.32 7.21 8.44 | 01 02 03 04 05 06 07 08 09 10 11 12 13 14 | 8.40 6.87 7.71 7.68 7.58 6.40 6.83 6.86 8.32 7.96 7.46 8.29 7.26 8.40 | 0.07 0.19 0.06 0.02 0.43 0.34 0.19 0.13 0.19 0.05 0.16 0.02 0.05 0.04 | 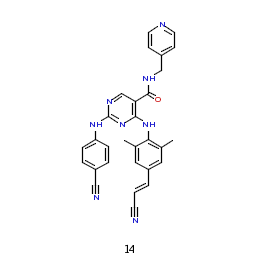 |
| 15 | 7.82 untested 7.78 7.83 7.81 6.31 untested untested 8.34 8.40 7.79 7.95 7.88 8.51 | 01 02 03 04 05 06 07 08 09 10 11 12 13 14 | 8.56 6.78 7.76 7.64 7.71 6.12 6.94 7.17 8.40 8.21 7.83 8.27 7.69 8.69 | 0.75 n/a 0.01 0.19 0.10 0.19 n/a n/a 0.06 0.19 0.05 0.32 0.19 0.19 | 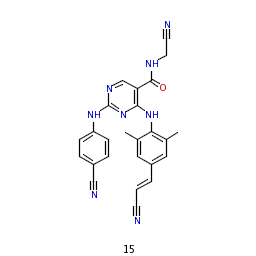 |
| 16 | 9.08 untested 7.85 7.71 6.95 5.66 untested untested 9.18 7.74 7.34 9.23 7.15 9.25 | 01 02 03 04 05 06 07 08 09 10 11 12 13 14 | 8.89 6.99 7.95 7.90 7.53 5.73 6.78 7.29 8.81 7.93 7.53 8.78 7.34 8.83 | 0.19 n/a 0.10 0.19 0.58 0.06 n/a n/a 0.37 0.19 0.19 0.45 0.19 0.43 | 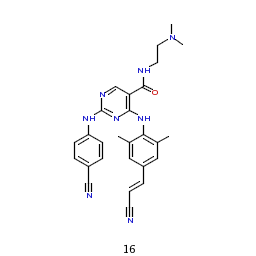 |
| 17 | 8.37 untested 7.80 8.10 7.95 6.13 untested untested untested 8.32 7.76 8.03 7.71 8.70 | 01 02 03 04 05 06 07 08 09 10 11 12 13 14 | 8.77 6.96 7.98 7.80 7.76 5.94 6.73 7.27 8.53 8.34 7.90 8.42 7.63 8.85 | 0.41 n/a 0.18 0.30 0.19 0.19 n/a n/a n/a 0.02 0.14 0.39 0.08 0.15 | 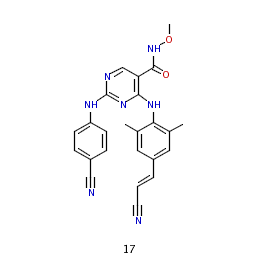 |
| 18 | 8.43 untested 7.85 8.46 7.81 4.60 untested untested 8.50 8.63 8.01 8.24 8.15 8.93 | 01 02 03 04 05 06 07 08 09 10 11 12 13 14 | 8.62 7.17 8.04 8.00 8.00 6.66 6.82 7.35 8.54 8.39 7.86 8.43 7.96 8.74 | 0.20 n/a 0.19 0.46 0.19 2.05 n/a n/a 0.03 0.24 0.16 0.18 0.19 0.19 | 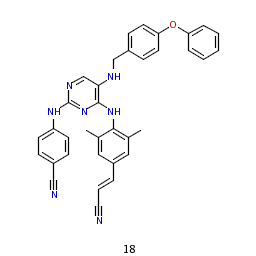 |
| 19 | 7.63 untested 6.93 6.91 6.12 4.97 untested untested 7.59 6.91 6.35 7.23 6.07 7.49 | 01 02 03 04 05 06 07 08 09 10 11 12 13 14 | 7.81 6.26 7.10 7.02 6.73 5.15 6.42 6.68 7.69 7.16 6.76 7.54 6.41 7.67 | 0.18 n/a 0.17 0.11 0.61 0.19 n/a n/a 0.10 0.25 0.40 0.30 0.34 0.19 | 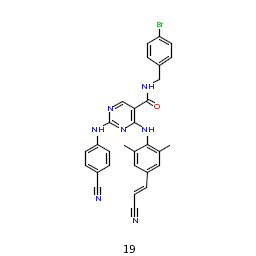 |
| 20 | 8.33 6.06 7.15 6.86 6.89 6.07 untested 5.67 7.97 7.03 6.70 7.30 7.74 8.81 | 01 02 03 04 05 06 07 08 09 10 11 12 13 14 | 7.84 6.25 7.13 7.05 7.14 5.71 7.26 6.67 7.71 7.59 6.89 7.51 7.55 8.62 | 0.49 0.19 0.02 0.19 0.25 0.35 n/a 1.00 0.26 0.57 0.19 0.21 0.19 0.19 | 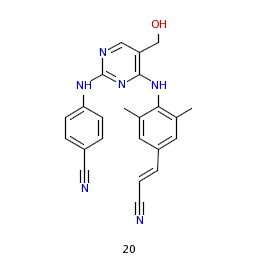 |
| 21 | 8.52 untested 8.51 8.36 8.44 6.46 untested untested 8.53 8.71 8.00 8.63 7.95 8.71 | 01 02 03 04 05 06 07 08 09 10 11 12 13 14 | 9.08 7.49 8.32 8.22 8.16 6.28 6.75 7.77 8.94 8.61 7.82 8.79 7.76 8.52 | 0.56 n/a 0.19 0.15 0.28 0.19 n/a n/a 0.41 0.10 0.19 0.16 0.19 0.19 | 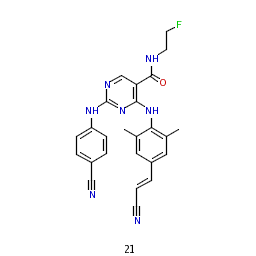 |
| 22 | 8.21 6.82 7.90 8.04 7.78 4.87 untested untested 8.53 8.52 8.21 8.33 8.10 8.82 | 01 02 03 04 05 06 07 08 09 10 11 12 13 14 | 8.68 7.09 7.95 7.85 7.89 6.00 6.35 7.24 8.54 8.33 8.03 8.46 7.91 8.63 | 0.48 0.27 0.06 0.19 0.10 1.13 n/a n/a 0.01 0.19 0.19 0.12 0.19 0.19 | 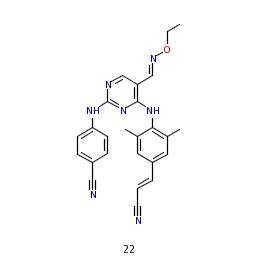 |
| 23 | 8.56 6.82 8.21 8.53 8.24 4.60 untested untested 8.96 8.84 9.05 8.47 8.44 9.01 | 01 02 03 04 05 06 07 08 09 10 11 12 13 14 | 8.78 7.07 8.30 8.34 8.43 6.31 6.38 8.00 8.77 8.64 8.86 8.65 8.25 8.82 | 0.22 0.25 0.09 0.19 0.19 1.71 n/a n/a 0.19 0.20 0.19 0.19 0.19 0.19 | 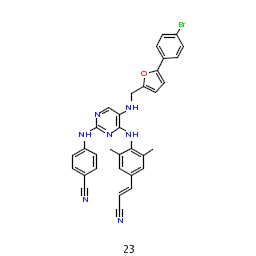 |
| 24 | 8.35 untested 7.88 7.95 8.51 5.81 untested untested 8.47 8.41 8.20 8.05 7.81 8.35 | 01 02 03 04 05 06 07 08 09 10 11 12 13 14 | 8.54 7.12 8.03 7.97 7.84 5.68 6.57 7.24 8.43 8.24 8.01 8.32 7.62 8.39 | 0.19 n/a 0.15 0.02 0.66 0.13 n/a n/a 0.04 0.17 0.19 0.27 0.19 0.04 | 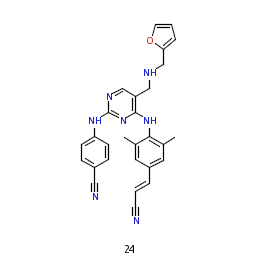 |
| 25 | 8.42 untested 7.64 7.64 7.68 5.50 untested untested 8.40 7.87 7.78 8.13 7.44 8.50 | 01 02 03 04 05 06 07 08 09 10 11 12 13 14 | 8.55 6.96 7.76 7.56 7.45 5.69 6.09 6.92 8.32 7.97 7.59 8.32 7.26 8.31 | 0.13 n/a 0.12 0.08 0.22 0.19 n/a n/a 0.08 0.10 0.19 0.19 0.19 0.19 | 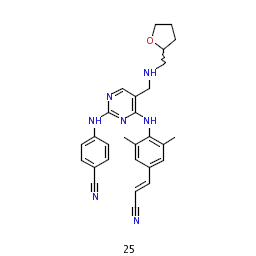 |
| 26 | 8.61 untested 8.09 8.02 7.57 5.67 untested untested 8.62 8.18 7.89 8.26 7.67 8.45 | 01 02 03 04 05 06 07 08 09 10 11 12 13 14 | 8.73 7.23 8.02 7.83 7.66 6.04 6.46 7.41 8.51 8.16 7.70 8.43 7.66 8.46 | 0.12 n/a 0.07 0.19 0.09 0.37 n/a n/a 0.11 0.02 0.19 0.17 0.01 0.02 | 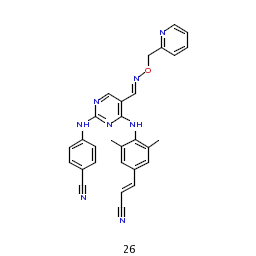 |
| 27 | 7.80 untested 6.91 7.04 7.42 5.38 untested untested 7.80 7.70 6.98 7.72 6.32 7.16 | 01 02 03 04 05 06 07 08 09 10 11 12 13 14 | 7.91 6.42 7.10 7.16 7.23 5.57 6.87 6.69 7.93 7.51 7.06 7.81 6.50 7.35 | 0.11 n/a 0.19 0.12 0.19 0.19 n/a n/a 0.13 0.19 0.07 0.09 0.19 0.19 | 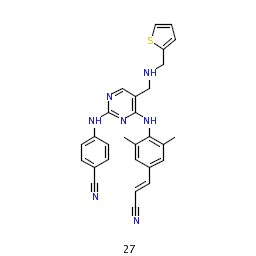 |
| 28 | 7.71 untested 6.87 6.64 6.28 4.97 untested untested 7.68 6.85 6.47 7.60 5.68 6.79 | 01 02 03 04 05 06 07 08 09 10 11 12 13 14 | 7.59 6.24 6.95 6.78 6.47 5.05 6.11 6.14 7.40 6.94 6.66 7.41 5.87 6.98 | 0.12 n/a 0.07 0.14 0.19 0.07 n/a n/a 0.28 0.08 0.19 0.19 0.19 0.19 | 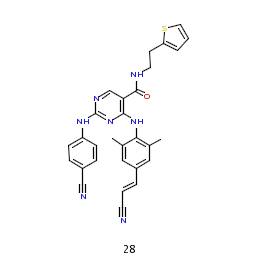 |
| 29 | 7.10 untested 6.46 6.63 6.60 4.60 untested untested 7.06 6.90 6.68 7.07 untested 6.88 | 01 02 03 04 05 06 07 08 09 10 11 12 13 14 | 7.28 6.29 6.65 6.54 6.42 5.55 6.20 6.09 7.14 6.79 6.49 7.15 6.19 6.95 | 0.18 n/a 0.19 0.09 0.19 0.95 n/a n/a 0.07 0.11 0.19 0.07 n/a 0.07 | 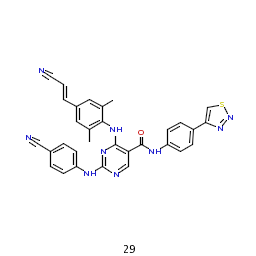 |
| 30 | 8.41 untested 7.07 7.45 7.15 4.60 untested untested 8.28 7.82 7.26 7.83 6.82 7.57 | 01 02 03 04 05 06 07 08 09 10 11 12 13 14 | 8.18 6.55 7.39 7.35 7.31 5.68 6.84 6.94 8.09 7.68 7.38 8.01 7.01 7.76 | 0.22 n/a 0.33 0.10 0.16 1.08 n/a n/a 0.19 0.14 0.12 0.17 0.19 0.19 | 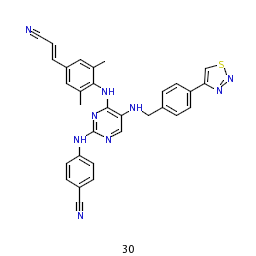 |
| 31 | 7.71 untested 6.90 6.97 6.22 4.60 untested untested 7.70 6.96 6.40 7.69 5.80 6.67 | 01 02 03 04 05 06 07 08 09 10 11 12 13 14 | 7.52 6.37 7.00 6.99 6.64 5.80 6.19 6.44 7.48 6.88 6.59 7.50 5.99 6.86 | 0.19 n/a 0.10 0.02 0.43 1.20 n/a n/a 0.23 0.08 0.19 0.19 0.19 0.19 | 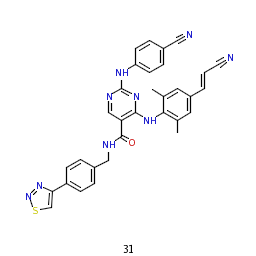 |
| 32 | 8.24 untested 7.61 7.64 7.15 4.65 untested untested 8.08 7.80 7.44 7.72 7.04 7.56 | 01 02 03 04 05 06 07 08 09 10 11 12 13 14 | 8.05 7.38 7.75 7.61 7.35 6.60 6.11 7.49 7.89 7.61 7.53 7.86 7.23 7.69 | 0.19 n/a 0.14 0.03 0.20 1.94 n/a n/a 0.19 0.19 0.09 0.15 0.19 0.12 | 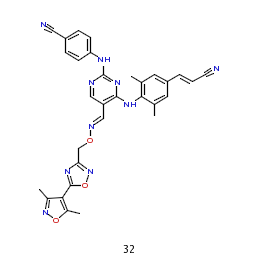 |
| 33 | 7.76 untested 7.34 7.59 7.16 5.21 untested untested 7.75 7.73 7.13 7.72 6.98 7.58 | 01 02 03 04 05 06 07 08 09 10 11 12 13 14 | 7.94 6.20 7.35 7.40 7.23 5.38 6.65 6.41 7.93 7.54 7.27 7.90 7.17 7.76 | 0.18 n/a 0.02 0.19 0.06 0.16 n/a n/a 0.19 0.19 0.15 0.18 0.19 0.19 | 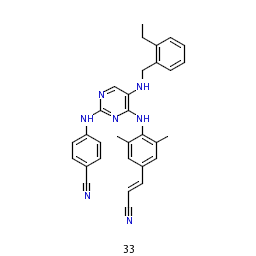 |
| 34 | 8.30 7.03 7.61 7.62 7.49 5.57 untested 6.11 8.36 7.86 7.21 8.22 7.04 7.57 | 01 02 03 04 05 06 07 08 09 10 11 12 13 14 | 8.27 6.84 7.66 7.53 7.38 5.76 6.43 6.33 8.10 7.82 7.40 8.23 7.22 7.76 | 0.03 0.19 0.05 0.10 0.11 0.19 n/a 0.22 0.26 0.05 0.19 0.02 0.19 0.19 | 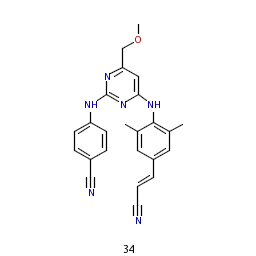 |
| 35 | 7.71 untested 7.07 7.02 5.83 4.60 untested untested 7.56 6.97 7.02 6.98 6.71 7.48 | 01 02 03 04 05 06 07 08 09 10 11 12 13 14 | 7.52 6.68 7.20 6.96 6.59 5.75 5.98 7.12 7.27 6.96 7.13 7.17 6.90 7.37 | 0.19 n/a 0.13 0.07 0.77 1.15 n/a n/a 0.30 0.01 0.11 0.19 0.19 0.11 | 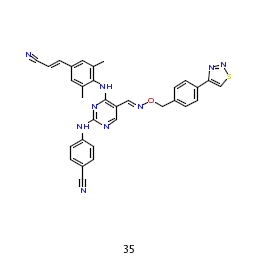 |
| 36 | 8.72 7.37 8.40 8.55 8.22 6.35 untested untested untested 8.74 8.45 8.53 8.35 8.76 | 01 02 03 04 05 06 07 08 09 10 11 12 13 14 | 8.91 7.30 8.33 8.36 8.26 6.16 6.37 7.23 8.88 8.61 8.26 8.83 8.16 8.74 | 0.19 0.07 0.07 0.19 0.04 0.19 n/a n/a n/a 0.13 0.19 0.30 0.19 0.02 | 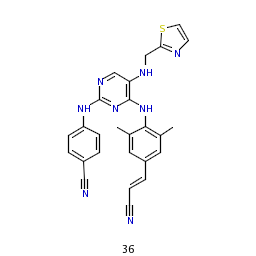 |
| 37 | 8.35 untested 7.82 7.97 7.71 5.45 untested untested untested 8.39 7.81 7.99 7.65 8.37 | 01 02 03 04 05 06 07 08 09 10 11 12 13 14 | 8.53 6.81 7.86 7.68 7.67 5.64 6.72 7.19 8.31 8.20 7.62 8.18 7.60 8.43 | 0.19 n/a 0.04 0.29 0.04 0.19 n/a n/a n/a 0.19 0.19 0.19 0.05 0.06 | 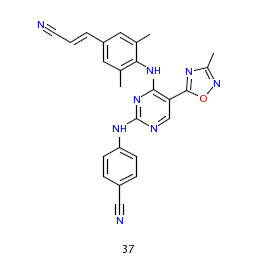 |
| 38 | 7.00 untested 6.27 5.93 5.04 4.60 untested untested untested 5.98 5.70 6.62 6.37 6.64 | 01 02 03 04 05 06 07 08 09 10 11 12 13 14 | 6.73 5.41 6.16 5.87 5.60 4.31 6.05 5.47 6.40 6.17 5.89 6.43 6.31 6.83 | 0.27 n/a 0.11 0.05 0.56 0.29 n/a n/a n/a 0.19 0.19 0.19 0.06 0.19 | 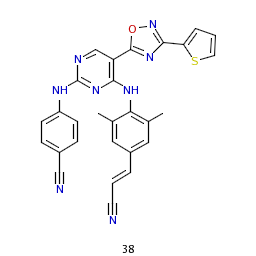 |
| 39 | 8.43 8.70 7.62 8.07 untested 5.57 untested untested untested untested untested untested untested untested | 01 02 03 04 05 06 07 08 09 10 11 12 13 14 | 8.38 7.31 7.81 7.71 7.54 5.76 6.76 6.95 8.24 7.97 7.51 8.16 7.48 7.91 | 0.05 1.40 0.19 0.36 n/a 0.19 n/a n/a n/a n/a n/a n/a n/a n/a | 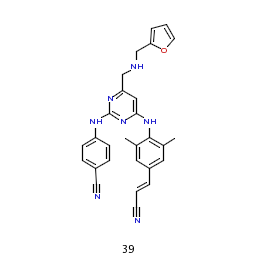 |
| 40 | 7.51 6.29 6.91 6.35 6.22 5.60 untested untested untested untested untested untested untested untested | 01 02 03 04 05 06 07 08 09 10 11 12 13 14 | 7.45 6.15 6.72 6.51 6.62 5.71 6.94 6.36 7.21 7.15 6.57 7.06 6.52 7.35 | 0.06 0.14 0.19 0.16 0.40 0.11 n/a n/a n/a n/a n/a n/a n/a n/a | 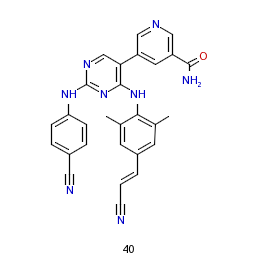 |
| 41 | 7.77 6.32 6.61 6.12 6.80 5.18 untested untested untested untested untested untested untested untested | 01 02 03 04 05 06 07 08 09 10 11 12 13 14 | 7.52 6.14 6.80 6.54 6.99 5.33 6.71 6.35 7.21 7.58 7.08 7.06 7.40 8.04 | 0.26 0.19 0.19 0.42 0.19 0.15 n/a n/a n/a n/a n/a n/a n/a n/a | 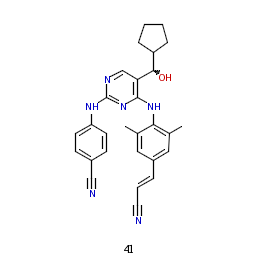 |
| 42 | 8.34 7.29 7.73 7.81 8.42 6.86 6.47 7.37 untested untested untested untested untested untested | 01 02 03 04 05 06 07 08 09 10 11 12 13 14 | 8.55 7.16 7.85 7.84 8.23 6.73 6.33 7.18 8.51 8.57 7.91 8.42 7.61 8.51 | 0.21 0.13 0.12 0.03 0.19 0.12 0.14 0.19 n/a n/a n/a n/a n/a n/a | 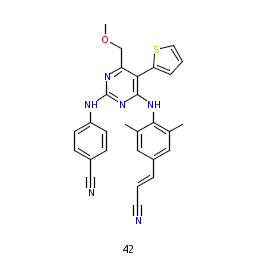 |
| 43 | 8.11 6.43 7.58 7.00 7.23 untested untested untested untested untested untested untested untested untested | 01 02 03 04 05 06 07 08 09 10 11 12 13 14 | 8.12 6.45 7.40 7.21 7.19 5.73 6.24 6.64 7.90 7.72 7.35 7.84 7.12 7.91 | 0.00 0.02 0.19 0.21 0.03 n/a n/a n/a n/a n/a n/a n/a n/a n/a | 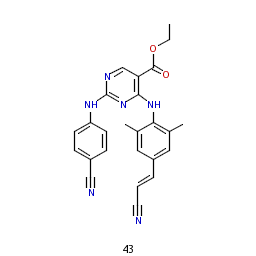 |
| 44 | 8.51 7.59 8.13 7.93 8.09 6.25 untested 6.07 untested 8.49 7.83 untested untested untested | 01 02 03 04 05 06 07 08 09 10 11 12 13 14 | 8.61 7.40 8.05 7.77 7.90 6.06 6.49 6.73 8.30 8.51 8.02 8.35 8.09 8.82 | 0.10 0.19 0.07 0.15 0.19 0.19 n/a 0.65 n/a 0.01 0.19 n/a n/a n/a | 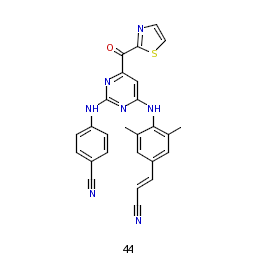 |
| 45 | 8.59 7.50 8.22 8.14 8.42 6.08 untested untested untested untested untested untested untested untested | 01 02 03 04 05 06 07 08 09 10 11 12 13 14 | 8.78 7.38 8.11 8.13 8.22 6.11 6.95 7.43 8.77 8.53 7.90 8.65 7.94 8.64 | 0.19 0.12 0.11 0.01 0.20 0.03 n/a n/a n/a n/a n/a n/a n/a n/a | 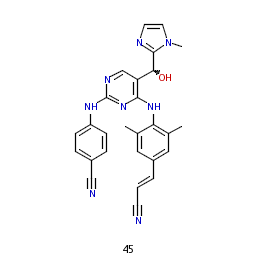 |
| 46 | 8.44 7.56 7.84 7.92 8.51 7.24 6.60 7.30 untested untested untested untested untested untested | 01 02 03 04 05 06 07 08 09 10 11 12 13 14 | 8.83 7.37 8.05 8.11 8.26 7.05 6.41 7.18 8.85 8.54 8.09 8.82 7.72 8.42 | 0.39 0.19 0.21 0.19 0.26 0.19 0.19 0.12 n/a n/a n/a n/a n/a n/a | 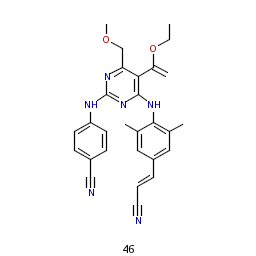 |
| 47 | 6.88 5.98 6.12 5.65 5.67 4.83 untested untested untested untested untested untested untested untested | 01 02 03 04 05 06 07 08 09 10 11 12 13 14 | 6.76 5.79 6.23 5.97 5.86 4.76 7.11 5.77 6.47 6.43 6.33 6.33 6.43 7.12 | 0.12 0.19 0.11 0.31 0.19 0.07 n/a n/a n/a n/a n/a n/a n/a n/a | 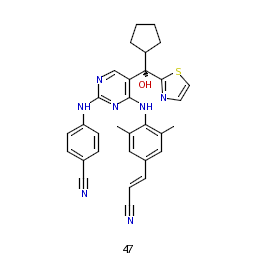 |
| 48 | 7.00 6.42 6.66 6.24 6.33 5.12 untested untested untested untested untested untested untested untested | 01 02 03 04 05 06 07 08 09 10 11 12 13 14 | 7.23 6.23 6.63 6.49 6.52 5.03 7.04 6.05 7.06 6.99 6.60 6.93 6.68 7.41 | 0.23 0.19 0.04 0.25 0.19 0.08 n/a n/a n/a n/a n/a n/a n/a n/a | 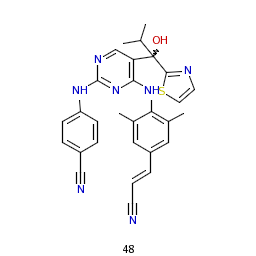 |
| 49 | 8.56 7.79 8.13 8.37 8.51 5.97 untested 7.72 untested untested untested untested untested untested | 01 02 03 04 05 06 07 08 09 10 11 12 13 14 | 8.92 7.67 8.31 8.27 8.32 6.16 7.50 7.69 8.84 8.70 8.07 8.66 7.95 8.93 | 0.36 0.12 0.18 0.11 0.19 0.19 n/a 0.03 n/a n/a n/a n/a n/a n/a | 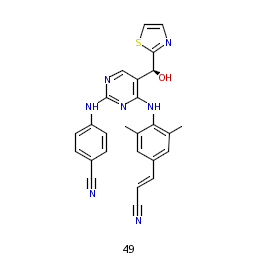 |
| 50 | 8.45 6.64 6.99 6.96 6.33 5.30 untested 6.10 untested untested untested untested untested untested | 01 02 03 04 05 06 07 08 09 10 11 12 13 14 | 7.54 6.74 7.11 6.94 6.81 5.71 6.75 6.29 7.34 7.24 6.87 7.31 6.98 7.43 | 0.91 0.10 0.12 0.01 0.48 0.40 n/a 0.19 n/a n/a n/a n/a n/a n/a | 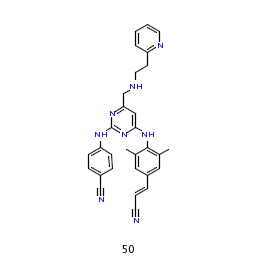 |
| 51 | 7.30 5.82 6.33 6.39 5.90 5.01 untested 4.98 untested untested untested untested untested untested | 01 02 03 04 05 06 07 08 09 10 11 12 13 14 | 7.12 5.73 6.41 6.20 6.12 5.07 6.00 5.26 6.87 6.69 6.53 6.96 6.65 7.39 | 0.18 0.08 0.08 0.19 0.23 0.06 n/a 0.27 n/a n/a n/a n/a n/a n/a | 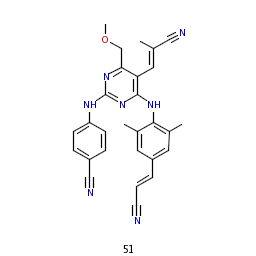 |
| 52 | 7.37 5.80 6.43 6.31 6.59 5.19 untested 5.67 untested untested untested untested untested untested | 01 02 03 04 05 06 07 08 09 10 11 12 13 14 | 7.18 5.77 6.57 6.39 6.40 5.38 6.14 5.86 6.96 6.84 6.74 7.00 6.92 7.50 | 0.19 0.04 0.14 0.08 0.19 0.19 n/a 0.19 n/a n/a n/a n/a n/a n/a | 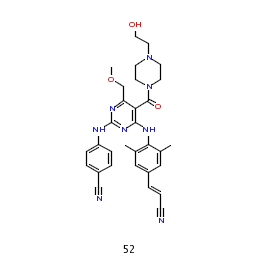 |
| 53 | 7.49 5.56 6.48 6.28 6.31 5.44 5.11 5.54 untested untested untested untested untested untested | 01 02 03 04 05 06 07 08 09 10 11 12 13 14 | 7.30 5.75 6.47 6.35 6.49 5.25 6.60 5.72 7.14 6.94 6.55 7.17 6.38 7.42 | 0.19 0.19 0.00 0.06 0.19 0.19 1.49 0.19 n/a n/a n/a n/a n/a n/a | 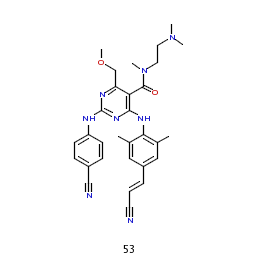 |
| 54 | 8.58 6.29 7.78 7.85 7.38 6.97 5.71 6.36 untested untested untested untested untested untested | 01 02 03 04 05 06 07 08 09 10 11 12 13 14 | 8.28 6.93 7.73 7.62 7.57 6.74 5.90 6.55 8.13 8.00 7.66 8.20 7.46 8.23 | 0.31 0.64 0.05 0.22 0.19 0.23 0.19 0.19 n/a n/a n/a n/a n/a n/a | 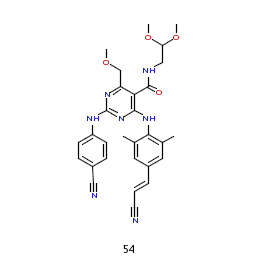 |
| 55 | 8.21 4.99 5.88 5.77 5.79 5.21 4.60 4.87 untested untested untested untested untested untested | 01 02 03 04 05 06 07 08 09 10 11 12 13 14 | 6.79 5.19 6.07 5.89 5.98 5.02 6.12 5.49 6.56 6.46 6.43 6.57 6.31 7.04 | 1.42 0.20 0.19 0.12 0.19 0.19 1.52 0.62 n/a n/a n/a n/a n/a n/a | 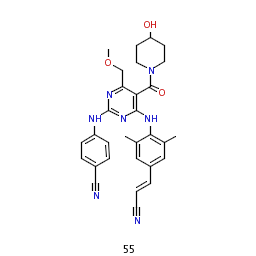 |
| 56 | 7.62 4.99 5.74 5.55 5.61 5.02 4.77 4.89 untested untested untested untested untested untested | 01 02 03 04 05 06 07 08 09 10 11 12 13 14 | 6.76 5.06 5.94 5.74 5.80 4.83 6.03 5.35 6.51 6.33 6.34 6.53 6.35 7.08 | 0.86 0.08 0.20 0.19 0.19 0.19 1.25 0.46 n/a n/a n/a n/a n/a n/a | 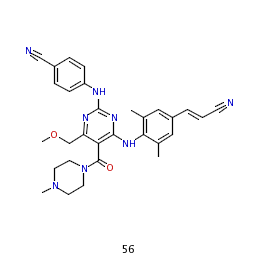 |
| 57 | 8.00 5.23 6.32 6.53 6.00 5.65 5.04 5.59 untested untested untested untested untested untested | 01 02 03 04 05 06 07 08 09 10 11 12 13 14 | 7.29 5.42 6.42 6.37 6.49 5.46 5.23 5.78 7.18 6.90 6.66 7.20 6.46 7.63 | 0.71 0.19 0.11 0.16 0.50 0.19 0.19 0.19 n/a n/a n/a n/a n/a n/a | 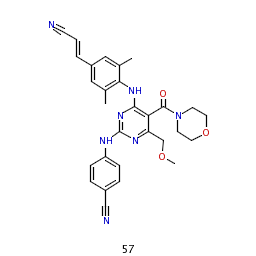 |
